# Macrophage TRIM21 Inhibition Ameliorates Murine Acute Pancreatitis via PHB2‐Mediated Mitochondrial Stabilization

**DOI:** 10.1002/advs.202517877

**Published:** 2026-01-22

**Authors:** Yansong Xu, Yuansong Sun, Xin Zhou, Kai Song, Chunlin Yin, Zhaohua Wang, Fei Xie, He Li

**Affiliations:** ^1^ Department of Emergency Medicine Qilu Hospital of Shandong University Jinan China; ^2^ Shandong Provincial Clinical Research Center For Emergency and Critical Care Medicine Chest Pain Center Institute of Emergency and Critical Care Medicine of Shandong University Qilu Hospital of Shandong University Jinan China; ^3^ Medical and Pharmaceutical Basic Research Innovation Center of Emergency and Critical Care Medicine Shandong Provincial Engineering Laboratory for Emergency and Critical Care Medicine Key Laboratory of Emergency and Critical Care Medicine of Shandong Province China's Ministry of Education Key Laboratory of Cardiopulmonary‐Cerebral Resuscitation Research of Shandong Province Qilu Hospital of Shandong University Jinan China; ^4^ NMPA Key Laboratory For Clinical Research and Evaluation of Innovative Drug Qilu Hospital of Shandong University Jinan China; ^5^ National Key Laboratory For Innovation and Transformation of Luobing Theory The Key Laboratory of Cardiovascular Remodeling and Function Research Chinese Ministry of Education Chinese National Health Commission and Chinese Academy of Medical Sciences Qilu Hospital of Shandong University Jinan China; ^6^ Department of Emergency Medicine the Second Affiliated Hospital of Anhui Medical University Hefei China

**Keywords:** acute pancreatitis, inflammation, macrophage, PHB2, TRIM21

## Abstract

Acute pancreatitis (AP) involves acinar cell death and severe inflammation. Although the E3 ubiquitin ligase TRIM21 regulates inflammation, its role in the pathogenesis of AP remains undefined. This study aims to explore the role of TRIM21 in regulating inflammation during AP. In this study, TRIM21 levels show a severity‐dependent increase in patients with AP, which is more than that in healthy controls. Consistently, increased TRIM21 expression is observed in the murine models of AP and exhibits spatial co‐localization with macrophages. Macrophage‐specific *Trim21* ablation mitigates pancreatic damage and systemic inflammation. Conversely, TRIM21 activation aggravates disease severity. Mechanistically, TRIM21 promotes K11‐linked ubiquitination and proteasomal degradation of PHB2, leading to mtDNA accumulation in the cytosol via impaired PHB2‐mediated mitophagy. Dysregulation of mtDNA homeostasis activates the cGAS‐STING axis, thereby intensifying inflammation during AP progression. Additionally, pharmacological inhibition of TRIM21 with quisinostat mitigates AP progression. Our findings reveal the critical role of TRIM21 in AP‐associated inflammation, providing a potential therapeutic strategy for inflammatory pancreatic diseases.

## Graphical abstract

Macrophage‐derived TRIM21 drives the progression of AP via ubiquitin‐proteasome‐mediated degradation of PHB2, leading to impaired PHB2‐mediated mitophagy. Therefore, accumulation of cytosolic mtDNA hyperactivates the cGAS‐STING signaling axis, thereby amplifying inflammatory cascades. The TRIM21‐PHB2‐mtDNA‐STING regulatory axis provides a mechanistic framework for macrophage‐based treatment of AP, wherein both AAV‐delivered sh*TRIM21* vectors and pharmacological inhibition of TRIM21 prevent inflammation‐driven disease exacerbation in AP.

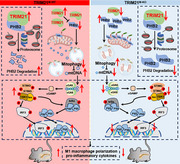

## 1 Introduction

Acute pancreatitis (AP), a prevalent gastrointestinal disorder, displays increasing incidence (approximately 3% annually), globally driven by modern risk factors, including advancing age, obesity, and alcohol abuse [1]. In addition, 20%–30% of patients with AP develop moderately severe/severe AP, with mortality rates reaching 30% in infected pancreatic necrosis [2]. Although premature intracellular activation of pancreatic enzymes, which triggers autodigestion and inflammation, is the pathognomonic mechanism in AP [3], the underlying molecular mechanisms remain incompletely defined. Current therapeutic options beyond supportive care remain limited. Insufficient mechanistic insight impedes treatment development, highlighting an urgent need for novel therapeutic targets.

The inflammatory cascade in AP principally originates from the dysregulated crosstalk between injured pancreatic acinar cells and recruited immune cells. As key first responders, innate immune cells, particularly macrophages and neutrophils, rapidly infiltrate into damaged tissues upon sensing acinar‐derived damage‐associated molecular patterns (DAMPs), initiating a defensive phagocytic clearance [4, 5]. Macrophages, which constitute the dominant immune population in the early phase of AP [4], clear the necrotic debris [6]. Paradoxically, this process activates macrophages, polarizing them toward a pro‐inflammatory phenotype with increased interleukin‐1 beta (IL‐1β) / tumor necrosis factor‐alpha (TNF‐α) secretion. The subsequent cytokine storm exacerbates parenchymal injury, expands necrosis, and disrupts inflammatory homeostasis. This self‐sustaining cascade may become systemic, resulting in systemic inflammatory response syndrome (SIRS). Unabated SIRS frequently progresses to multiple organ dysfunction syndrome (MODS), which is the primary driver of mortality in AP [7, 8].

The tripartite motif (TRIM) family of E3 ubiquitin ligases orchestrates diverse cellular processes, including innate immunity, inflammatory regulation, autophagy, and tumorigenesis [9, 10]. Accumulating evidence establishes their critical role in modulating inflammation, designating TRIM proteins as master regulators in inflammatory disorders [11]. Specifically, TRIM21 (also known as Ro52/SSA) is an autoantigen implicated in systemic lupus erythematosus and Sjögren's syndrome [12]. This molecule plays a key role in resolving inflammation [13] and controlling the autophagic flux [14]. Crucially, macrophage‐intrinsic TRIM21 activity was shown to exacerbate inflammation in myocardial infarction [13] and obesity‐associated pathologies [15]. TRIM21 promotes M1 macrophage polarization via the phosphatidylinositol 3‐kinase (PI3K)/protein kinase B (PKB/AKT) signaling pathway, leading to increased production of pro‐inflammatory cytokines (e.g., IL‐6, TNF‐α) and impairing cardiac repair in myocardial infarction [13]. In obesity, TRIM21 enhances the ubiquitination and degradation of the Von Hippel‐Lindau (VHL) tumor suppressor, thereby stabilizing hypoxia‐inducible factor 1α (HIF‐1α) and driving IL‐1β production, which in turn drives chronic inflammation and metabolic dysfunction [15]. Despite its established role in inflammatory cascades, the mechanistic contribution of TRIM21 to the pathogenesis of AP remains underexplored.

Prohibitin 2 (PHB2), a highly conserved and ubiquitously expressed protein localized on the mitochondrial inner membrane [16], critically sustains mitochondrial homeostasis. When mitochondria are damaged, PHB2 acts as a mitophagy receptor, directly recruiting microtubule‐associated protein 1 light chain 3 beta (LC3B) [17], and simultaneously modulating the inner membrane protease presenilin associated rhomboid like (PARL) to stabilize PTEN induced kinase 1 (PINK1) [18], thereby synergistically promoting PINK1‐parkin RBR E3 ubiquitin protein ligase (Parkin)‐mediated mitophagy to maintain mitochondrial integrity and prevent pathological cascades triggered by cytoplasmic leakage of mitochondrial components, especially mitochondrial DNA (mtDNA) [19].

Here, we demonstrated that TRIM21 upregulation in pancreatic macrophages during AP disrupts mitophagy by modulating PHB2, leading to the pathological accumulation of mtDNA in the cytosol. Aberrant mtDNA release activates the cyclic GMP‐AMP synthase (cGAS)‐stimulator of interferon genes (STING) signaling, driving persistent pancreatic and systemic inflammation. Crucially, genetic deletion or pharmacological inhibition of macrophage TRIM21 significantly mitigates the severity of AP. These findings elucidate a novel pathological mechanism and reveal promising therapeutic strategies focused on the TRIM21‐PHB2‐cGAS/STING axis.

## 2 Results

### 2.1 Elevated Levels of Macrophage‐Derived TRIM21 were Associated with AP

**FIGURE 1:**
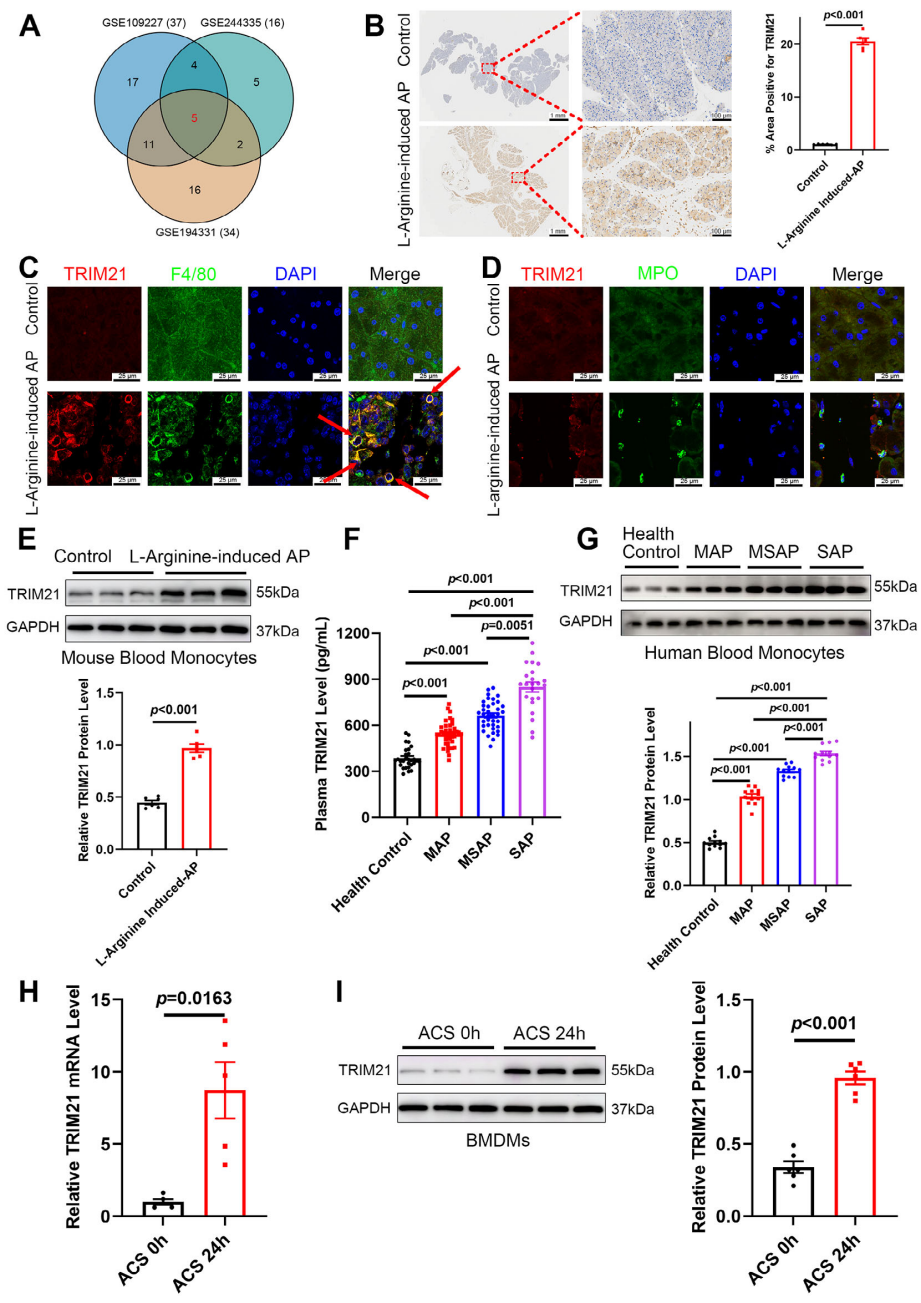
TRIM21 expression was elevated in acute pancreatitis (AP). (A) Cross‐dataset analysis of TRIM family gene expression using three independent Gene Expression Omnibus (GEO) datasets. (B) Immunohistochemical (IHC) staining for TRIM21 in the pancreas from L‐arginine‐treated and saline‐treated mice (scale bar: 100 µm; *n* = 6). (C, D) Co‐immunofluorescence of TRIM21 (red) with F4/80 (green; macrophage marker) or MPO (green; neutrophil marker). Cell nuclei were stained with DAPI (scale bar: 25 µm; *n* = 5). (E) Representative Western blotting images (upper panel) and quantification (lower panel) of the expression levels of TRIM21 protein in circulating monocytes from L‐arginine‐treated and saline‐treated mice (*n* = 6). (F) The plasma levels of TRIM21 in healthy controls (*n* = 29) and those with mild AP (*n* = 41), moderately severe AP (*n* = 37), and severe AP (*n* = 23). (G) Representative Western blotting images (upper panel) and quantification (lower panel) of TRIM21 levels in circulating monocytes from patients with AP (*n* = 12 per subtype) and healthy controls (*n* = 12). (H) RT‐qPCR analysis of the mRNA levels of TRIM21 (*n* = 5) and (I) Western blotting of the protein levels of TRIM21 (*n* = 6) in bone marrow‐derived macrophages (BMDMs) treated with acinar cell supernatants (ACS). Data are expressed as mean ± SEM (B, E–I), and statistical analyses were conducted using Welch's t‐test (B, H), Student's t‐test (E, I), Kruskal‐Wallis test with Dunn's post hoc test (F), and ANOVA with Tukey HSD post hoc test (G).

Given the well‐established involvement of the TRIM family of proteins in inflammation, we analyzed datasets from the Gene Expression Omnibus (GEO) to identify dysregulated TRIM genes (*p* < 0.05). Cross‐analysis of three independent datasets revealed five dysregulated TRIM genes: *Trim8, Trim21, Trim37, Trim41, and Trim65* (Figure 1A). To determine the most relevant candidate in AP pathogenesis, we performed qPCR validation on pancreatic tissues from our L‐arginine‐induced murine AP model. The results revealed that the mRNA levels of *Trim8, Trim37, Trim41, and Trim65* were very low in pancreatic tissues from the L‐arginine‐induced murine AP model, whereas *Trim21* exhibited the most pronounced and abundant upregulation (Figure S1A). Based on this distinct expression profile and its established inflammatory functions, we selected *Trim21* for further investigation into its functional role in AP. To validate the pathological significance of *Trim21* identified above, we examined its expression in the L‐arginine‐induced AP model. Consistent with the screening data, TRIM21 expression was significantly increased in pancreatic tissues (Figure 1B). Since macrophages and neutrophils constitute the predominant types of infiltrating immune cells in the early phase of AP, we conducted immunofluorescence staining to determine cellular localization. In the pancreas of mice with L‐arginine‐induced AP, TRIM21 expression was predominantly enriched in macrophages, exhibiting stronger co‐localization signals with the macrophage marker F4/80 than with the neutrophil marker myeloperoxidase (MPO) (Figure 1C,D). Consistently, we observed TRIM21 upregulation in circulating monocytes from these mice (Figure 1E). To assess clinical relevance, we recruited 101 patients with AP and 29 age‐matched healthy volunteers, who showed no statistically significant differences in baseline characteristics, including age, sex, body mass index (BMI), and comorbidities (hypertension and diabetes mellitus; Table S1). In plasma samples and circulating monocytes, TRIM21 expression increased with AP severity (Figure 1F,G), and the elevation of plasma samples showed no statistically significant sex‐specific difference (Figure S1B). Modeling the AP microenvironment in vitro, we stimulated bone marrow‐derived macrophages (BMDMs) with acinar cell supernatants (ACS), observing synchronous upregulation of TRIM21 mRNA (Figure 1H) and protein (Figure 1I). This protein upregulation was also comparable in BMDMs from both male and female mice, with no significant sex‐specific differences observed (Figure S1C). These findings indicate that macrophage‐derived TRIM21 may regulate AP by modulating inflammation.

### 2.2 Macrophage‐Specific Trim21 Deficiency Suppressed the Inflammatory Response and Mitigated the Development of AP

**FIGURE 2:**
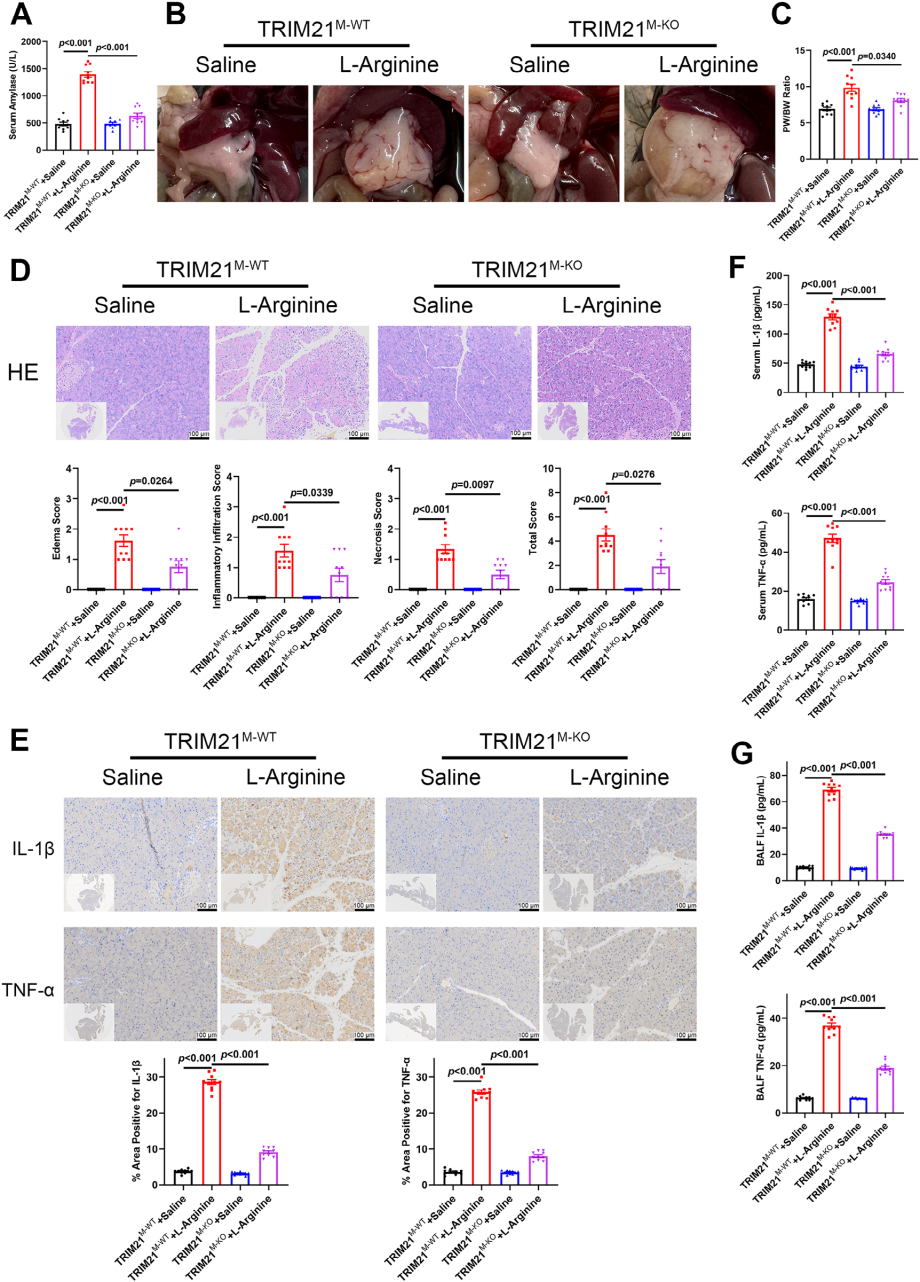
Macrophage *Trim21* deficiency attenuated L‐arginine‐induced AP. (A–G) *Trim21^M‐KO^* and *Trim21^M‐WT^* mice were treated with either saline or L‐arginine for 72 h (*Trim21^M‐WT^* + Saline, *Trim21^M‐WT^* + L‐arginine, *Trim21^M‐KO^* + Saline, *Trim21^M‐KO^* + L‐arginine; *n* = 10 per group). (A) Serum amylase levels. (B) Representative gross morphology of the pancreas. (C) Pancreas‐to‐body weight ratio. (D) Hematoxylin and eosin (H&E) staining (upper panel) and histopathological scoring (lower panel) of the pancreatic tissue. (E) Representative images (upper panel) and quantification (lower panel) of IHC staining for interleukin‐1 beta (IL‐1β) / tumor necrosis factor‐alpha (TNF‐α) in the pancreatic tissue. (F, G) IL‐1β and TNF‐α levels in serum samples and bronchoalveolar lavage fluid (BALF). Data are expressed as mean ± SEM (A, C–G), and statistical analyses were conducted using ANOVA with Tukey HSD post hoc test (A, TNF‐α in E), Welch's ANOVA with Games‐Howell post hoc test (C, IL‐1β in E, F, G), and Kruskal‐Wallis test with Dunn's post hoc test (D).

To further substantiate the effect of macrophage TRIM21 in the development of AP, we established macrophage‐specific *Trim21* knockout mice (*Trim21^M‐KO^*) and an AP model induced by intraperitoneal injection of L‐arginine. L‐arginine significantly increased serum amylase level in *Trim21^M‐WT^* mice, whereas *Trim21^M‐KO^* littermates exhibited markedly reduced levels (Figure 2A). Gross morphological assessment revealed pronounced pancreatic edema in *Trim21^M‐WT^* mice, characterized by significantly elevated pancreas‐to‐body weight ratios (Figure 2B,C). This pathological edema was substantially attenuated in *Trim21^M‐KO^* mice. Consistent with these findings, Hematoxylin and eosin (H&E)‐based histopathological scoring confirmed that *Trim21* knockout significantly mitigated pancreatic injury in L‐arginine‐treated AP mice compared to controls, manifesting with a marked attenuation of interstitial edema, diminished inflammatory cell infiltration, and reduced acinar cell necrosis (Figure 2D). Since excessive inflammatory response plays a critical role in the pathogenesis of AP, we measured the levels of pro‐inflammatory cytokines (IL‐1β and TNF‐α) in the murine models of AP. Immunohistochemical (IHC) analysis of pancreatic tissues revealed significantly lower protein expression levels of IL‐1β and TNF‐α in *Trim21^M‐KO^* mice compared to *Trim21^M‐WT^* mice (Figure 2E). Since acute lung injury is a critical extra‐pancreatic manifestation of AP, pro‐inflammatory cytokines were measured not only in serum samples but also in bronchoalveolar lavage fluid (BALF) to ensure the comprehensive assessment of systemic inflammation (Figure 2F,G). Consistent increases in cytokine levels were observed in serum and BALF following treatment with L‐arginine, with significantly higher levels observed in *Trim21^M‐WT^* mice. Conversely, *Trim21^M‐KO^* mice exhibited markedly decreased cytokine levels compared to *Trim21^M‐WT^* littermates. Furthermore, to assess potential sex‐specific effects, we established the L‐arginine‐induced AP model in age‐matched *Trim21^M‐KO^* and *Trim21^M‐WT^* mice of both sexes. H&E‐based histopathological scoring revealed a significant amelioration of pancreatic injury in *Trim21^M‐KO^* AP mice compared to their *Trim21^M‐WT^* counterparts, with no significant sex‐specific difference observed (Figure S2A).

In vitro, using BMDMs isolated from *Trim21^M‐WT^* mice and *Trim21^M‐KO^* mice indicated that genetic ablation of *Trim21* reversed ACS‐induced upregulations of iNOS (an M1 macrophage marker) and multiple inflammasome‐associated proteins, including NOD‐like receptor family pyrin domain containing 3 (NLRP3), pro‐cysteinyl aspartate specific proteinase‐1 (pro‐caspase‐1), apoptosis‐associated speck‐like protein containing a CARD (ASC), pro‐IL‐1β, and TNF‐α. Concomitantly, this intervention rescued the expression of Arg1 (an M2 macrophage marker), which was suppressed by ACS (Figure S2B,C). These experimental findings strongly align with well‐characterized pathological mechanisms, whereby pro‐inflammatory cytokines originating from macrophages promote the progression of AP [20]. Taken together, our findings conclusively suggest that macrophage‐specific TRIM21 deficiency attenuates inflammatory response and mitigates the development of AP.

### 2.3 Vilazodone‐Induced TRIM21 Activation Enhanced the Inflammatory Response and Exacerbated the Development of AP

**FIGURE 3:**
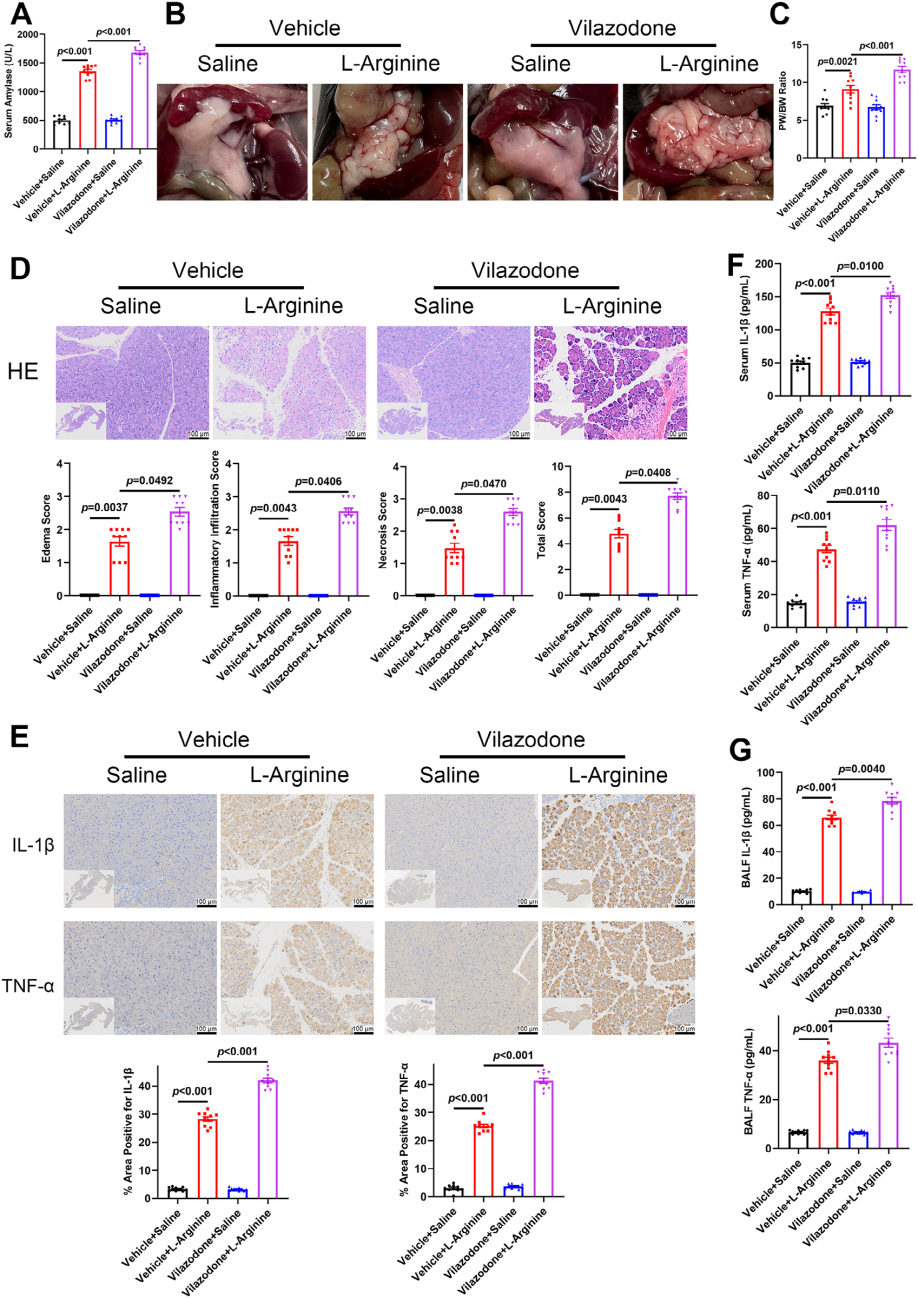
Vilazodone‐mediated activation of TRIM21 exacerbated L‐arginine‐induced AP. (A‐G) *Trim21^M‐WT^* mice pretreated with vehicle or vilazodone intraperitoneally received saline or L‐arginine for 72 h (Vehicle + Saline, Vehicle + L‐arginine, Vilazodone + Saline, Vilazodone + L‐arginine; *n* = 10 per group). (A) Serum amylase levels. (B) Representative gross morphology of the pancreas. (C) Pancreas‐to‐body weight ratio. (D) H&E staining (upper panel) and histopathological scores (lower panel) of the pancreatic tissue. (E) Representative images (upper panel) and quantification (lower panel) of IHC staining for IL‐1β and TNF‐α in pancreatic tissue. (F, G) IL‐1β and TNF‐α levels in serum samples and BALF. Data are expressed as mean ± SEM (A, C–G), and statistical analyses were conducted using ANOVA with Tukey HSD post hoc test (A, C), Kruskal‐Wallis test with Dunn's post hoc test (D), and Welch's ANOVA with Games‐Howell post hoc test (E–G).

Vilazodone, a direct activator of TRIM21 E3 ligase function [21], was used to pharmacologically activate TRIM21 in *Trim21^M‐WT^* mice during AP. Vilazodone‐induced TRIM21 activation significantly elevated the serum levels of amylase in L‐arginine‐treated mice (Figure 3A). Compared to controls, vilazodone‐treated mice exhibited pronounced pancreatic edema and increased pancreas‐to‐body weight ratios (Figure 3B,C). H&E staining indicated that vilazodone significantly exacerbated pancreatic injury in L‐arginine‐treated AP mice compared to controls (Figure 3D). IHC and ELISA confirmed aggravated local and systemic inflammation, evidenced by increased levels of IL‐1β and TNF‐α in vilazodone‐treated mice with AP (Figure 3E–G). To further confirm that the aggravating effect of vilazodone was primarily mediated through TRIM21, we pretreated *Trim21^M‐KO^* and *Trim21^M‐WT^* mice intraperitoneally with vehicle or vilazodone, followed by L‐arginine administration for 72 h. Histopathological scoring of H&E‐stained sections revealed that vilazodone pretreatment significantly exacerbated pancreatic injury in *Trim21^M‐WT^* AP mice (Figure S3A). In contrast, this exacerbating effect was abolished in *Trim21^M‐KO^* AP mice, where vilazodone treatment did not cause a statistically significant increase in injury scores compared to the control group (Figure S3A). These data demonstrate that vilazodone aggravates AP in a TRIM21‑dependent manner. In vitro, upon ACS stimulation, BMDMs from *Trim21^M‐WT^* mice transfected with an adenovirus carrying *Trim21* (Ad‐*Trim21*) showed upregulation of pro‐inflammatory mediators (iNOS, NLRP3, pro‐caspase‐1, ASC, pro‐IL‐1β, and TNF‐α) and concomitant suppression of Arg1 expression, compared to Ad‐*NC*‐transfected controls (Figure S3B,C). Collectively, pharmacological activation of TRIM21 amplified macrophage‐driven inflammation and accelerated the progression of AP.

### 2.4 Macrophage TRIM21‐PHB2 Axis Regulated the Inflammatory Response Through Mitophagy/mtDNA‐Driven cGAS‐STING Activation

**FIGURE 4:**
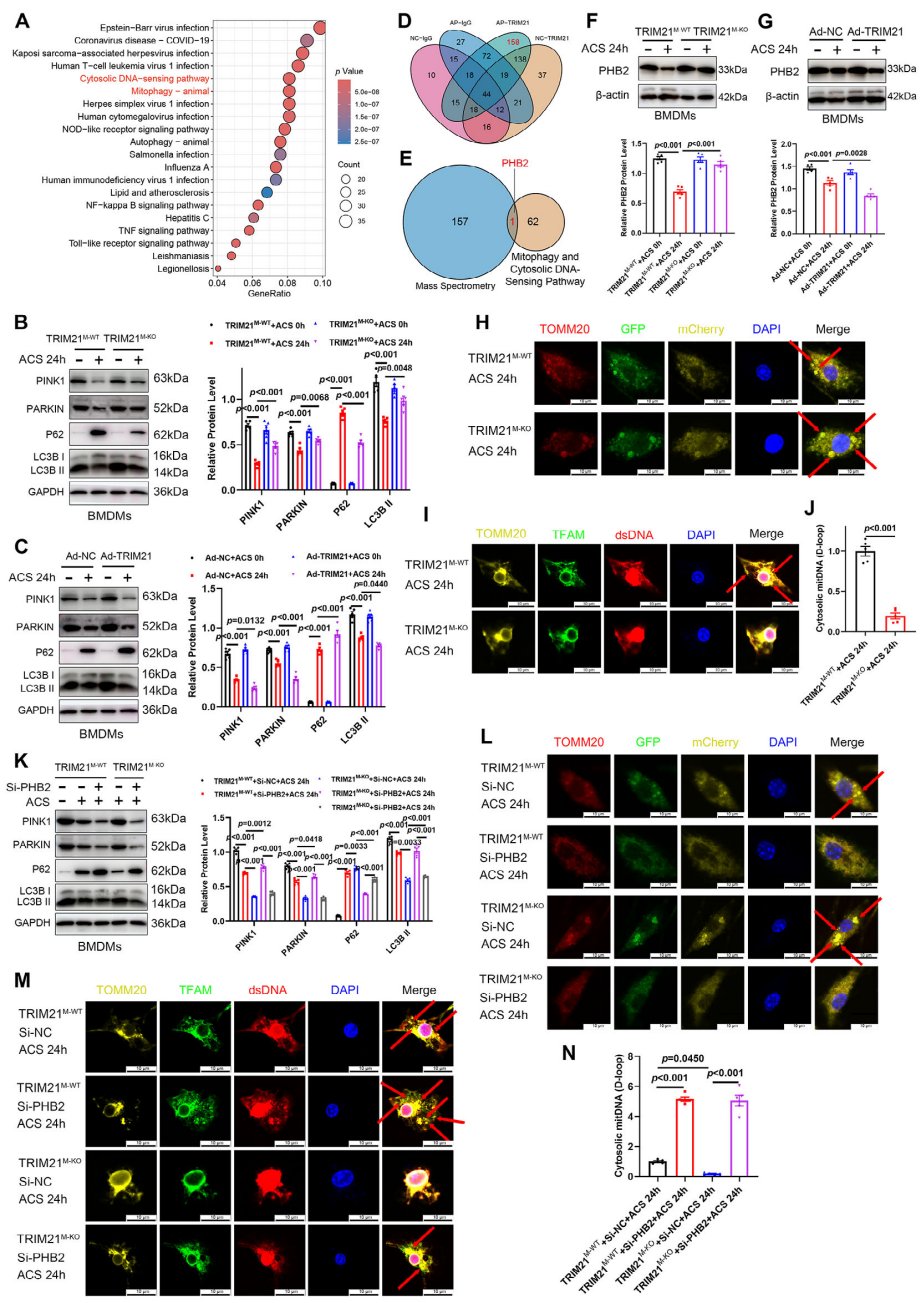
Macrophage TRIM21 regulates PHB2‐mediated cGAS/STING signaling pathways. (A) Kyoto Encyclopedia of Genes and Genomes (KEGG) analysis of differentially expressed proteins (DEPs) in pancreatic tissues from L‐arginine‐induced *Trim21^M‐WT^* and *Trim21^M‐KO^* mice (*n* = 3). The bubble diagram represents the number of genes under a specific term. The color of the dots represents the *p*‐value. (B, C) Representative Western blotting images and quantification of the expression levels of mitophagy‐associated proteins (PINK1, Parkin, p62, and LC3B) in BMDMs from *Trim21^M‐WT^* and *Trim21^M‐KO^* mice, or *Trim21^M‐WT^* mice transfected with an adenovirus carrying *Trim21* (Ad‐*Trim21*) and then stimulated with ACS for 12 h (*n* = 5). (D) Co‐immunoprecipitation (co‐IP) with liquid chromatography‐tandem mass spectrometry (LC‐MS/MS) analysis of pancreatic tissues from L‐arginine‐induced AP and saline‐induced control, with anti‐TRIM21 antibody or IgG antibody (*n* = 3). (E) Intersection analysis of the DEPs associated with mitophagy and cytosolic DNA‐sensing pathways with the exclusive LC‐MS/MS signature proteins. (F, G) Western blotting of PHB2 in BMDMs from *Trim21^M‐WT^* and *Trim21^M‐KO^* mice, or *Trim21^M‐WT^* mice transfected with Ad‐*Trim21* and then stimulated with ACS for 12 h (*n* = 5). (H) Representative immunofluorescence images of mitophagic events in BMDMs from *Trim21^M‐WT^* or *Trim21^M‐KO^* mice after 12 h of treatment with ACS. Mitochondria (TOMM20, red) colocalizing with the autophagic marker LC3B (visualized using adenoviral mCherry‐GFP‐LC3B fusion protein) were identified as mitophagic events (indicated by the red arrows; scale bar: 10 µm; *n* = 5). (I) Representative immunofluorescence images of cytosolic mtDNA accumulation in BMDMs from *Trim21^M‐WT^* or *Trim21^M‐KO^* mice after 12 h of treatment with ACS. Areas where TFAM (mitochondrial transcription factor A, green) and double‐stranded DNA (dsDNA, red) signals overlapped, but did not co‐localize with mitochondria (TOMM20, yellow) or the nucleus (DAPI, blue), were identified as cytosolic mtDNA (indicated by the red arrows; scale bar: 10 µm; *n* = 5). (J) RT‐qPCR of cytosolic mtDNA in BMDMs from *Trim21^M‐WT^* or *Trim21^M‐KO^* mice after 12 h of treatment with ACS (*n* = 5). (K) Representative Western blot images (left panel) and quantification (right panel) of mitophagy‐associated protein levels (PINK1, Parkin, p62, and LC3B) in BMDMs from *Trim21^M‐WT^* or *Trim21^M‐KO^* mice following transfection with *PHB2* siRNA (si‐*Phb2*) and subsequent 12‐h ACS treatment (*n* = 5). (L) Representative immunofluorescence images of mitophagic events in BMDMs from *Trim21^M‐WT^* or *Trim21^M‐KO^* mice after transfection with *Phb2* siRNA (si‐*Phb2*) and 12 h of treatment with ACS. TOMM20 (red) colocalizing with the autophagic marker LC3B (visualized using adenoviral mCherry‐GFP‐LC3B fusion protein) was identified as a mitophagic event (indicated by the red arrows; scale bar: 10 µm; *n* = 5). (M) Representative immunofluorescence images (indicated by the red arrows; scale bar: 10 µm; *n* = 5) and (N) RT‐qPCR (*n* = 5) of cytosolic mtDNA in BMDMs from *Trim21^M‐WT^* or *Trim21^M‐KO^* mice after transfection with si‐*Phb2* and 12 h of treatment with ACS. Data are expressed as mean ± SEM (B, C, F, G, J, K, N). Statistical analyses were conducted using ANOVA with Tukey HSD post hoc test (indicators other than p62 in B, C, F, G, K, N), Welch's ANOVA with Games‐Howell post hoc test (p62 in B), and Student's t‐test (J).

To investigate the molecular mechanism of macrophage TRIM21 in AP, we conducted proteomic profiling on pancreatic tissues from L‐arginine‐induced *Trim21^M‐WT^* and *Trim21^M‐KO^* mice, identifying 630 differentially expressed proteins (DEPs) upon macrophage *Trim21* ablation (Table S2). Kyoto Encyclopedia of Genes and Genomes (KEGG) analysis revealed TRIM21‐associated enrichment in viral infection, cytosolic DNA‐sensing pathways, mitophagy, and inflammatory pathways (Figure 4A). The lack of linkage between the top‐ranking viral infection pathways and AP prompted a shift in focus to the mitophagy and cytosolic DNA‐sensing pathways, due to their pivotal role in driving pro‐inflammatory responses [20, 22]. To confirm whether TRIM21 affects these pathways, we conducted in vitro experiments using BMDMs. We found that macrophage *Trim21* deletion reversed the ACS‐induced depletion of PINK1, Parkin, and LC3B‐II, attenuated p62 accumulation (Figure 4B), and suppressed the expression of cGAS‐STING pathway components (Figure S4A,B). Conversely, TRIM21 overexpression exacerbated the ACS‐induced expression dynamics of key components across both pathways (Figure 4C; Figure S4A,C). Collectively, these results indicate that TRIM21 regulates inflammatory responses mediated by mitophagy and the cGAS‐STING pathway in macrophages following ACS stimulation. To determine whether the expression of proteins in these pathways exhibits sex‐specific differences, we first performed in vitro studies using BMDMs isolated from male and female *Trim21^M‐WT^* mice. Following ACS stimulation, Western blot analysis revealed comparable changes in key mitophagy proteins (PINK1, Parkin, p62, and LC3B‐II), with no significant differences observed between sexes (Figure S4D,E). Furthermore, we established the L‐arginine‐induced AP model in age‐matched *Trim21^M‐KO^* and *Trim21^M‐WT^* mice of both sexes. Immunofluorescence analysis of pancreatic tissues revealed that the expression of the mitophagy marker PINK1 was significantly increased in macrophages of *Trim21^M‐KO^* AP mice compared to their *Trim21^M‐WT^* counterparts, with no significant sex‐specific difference observed (Figure S4F,G). Taken together, these data indicate an absence of sex‐dependent variation in the activation of the TRIM21‐regulated mitophagy.

Subsequently, to identify downstream targets mediating TRIM21 function in macrophages, we conducted co‐immunoprecipitation (co‐IP) coupled with liquid chromatography‐tandem mass spectrometry (LC‐MS/MS) using a TRIM21‐specific antibody, revealing 158 unique interactors in pancreatic tissues after induction with L‐arginine (Figure 4D; Table S3). An intersection with the 63 DEPs associated with mitophagy and cytosolic DNA‐sensing pathways identified PHB2 as the sole overlapping protein (Figure 4E). Proteomic analysis revealed a negative correlation between PHB2 and TRIM21 levels. To validate these findings in vitro, we examined PHB2 expression in ACS‐challenged BMDMs. *Trim21* deficiency substantially increased PHB2 levels, whereas *Trim21* overexpression suppressed its levels in ACS‐challenged BMDMs (Figure 4F,G), without altering the mRNA expression of *Phb2* (Figure S4H,I), indicating post‐transcriptional regulation. Given that PHB2 maintains mitochondrial homeostasis via PINK1‐Parkin‐mediated mitophagy—a process known to prevent mtDNA leakage and subsequent cGAS‐STING activation [18, 19, 23]—we hypothesized that by degrading PHB2, TRIM21 inhibits mitophagy, thereby promoting mtDNA leakage and ultimately driving inflammation. Consistent with this hypothesis, immunofluorescence imaging showed that *Trim21* knockout reversed ACS‐induced impairment of mitophagic flux (Figure 4H). Furthermore, both immunofluorescence and qPCR analyses (D‐loop fragment) confirmed that cytosolic mtDNA levels were significantly decreased after *Trim21* knockout (Figure 4I,J). Collectively, these results establish that TRIM21 suppresses PINK1‐Parkin‐dependent mitophagy to promote mtDNA leakage and cGAS‐STING activation.

To confirm whether PHB2 mediates TRIM21‐dependent regulation of mitophagy and cytosolic mtDNA accumulation, we transfected *Phb2* siRNA (si*‐Phb2*) into BMDMs. After ACS stimulation, *Phb2* knockdown reversed *Trim21* deficiency‐induced upregulation of mitophagy components (PINK1, Parkin, and LC3B‐II) and restored p62 accumulation (Figure 4K). Concurrently, immunofluorescence indicated that *Phb2* knockdown reversed enhanced mitophagosome formation in *Trim21^M‐KO^* BMDMs (Figure 4L). Immunofluorescence and qPCR analyses consistently revealed that *Phb2* knockdown reversed *Trim21* deficiency‐mediated suppression of mtDNA accumulation in the cytosol (Figure 4M,N). Furthermore, *Phb2* knockdown rescued the inhibitory effects of *Trim21* knockout on cGAS‐STING signaling (Figure S4J,K). Collectively, macrophage TRIM21 governs cytosolic mtDNA homeostasis by inhibiting PHB2‐mediated mitophagy, thereby activating cGAS‐STING‐driven inflammatory cascades in AP.

To investigate the generalizability of the TRIM21‐PHB2–mitophagy axis beyond the L‐arginine model (which may favor this axis due to direct mitochondrial damage [24]), we employed a caerulein‐induced AP model that operates through a distinct, receptor‐mediated secretory mechanism. Consistent with our earlier findings, macrophage‑specific *Trim21* knockout significantly mitigated pancreatic injury in this model, with reduced edema, inflammatory infiltration, and acinar necrosis (Figure S5A). Immunofluorescence analysis further confirmed that *Trim21* deficiency increased expression of the mitophagy initiator PINK1 (Figure S5B) and decreased p‐STING (Figure S5C) in pancreatic macrophages. These results demonstrate the protective effect of macrophage *Trim21* ablation and its associated molecular signature in two distinct mouse AP models: one induced by L‐arginine and the other by caerulein.

### 2.5 TRIM21 Mediated PHB2 Degradation via K11‐Ubiquitin‐Dependent Proteasomal Targeting

**FIGURE 5:**
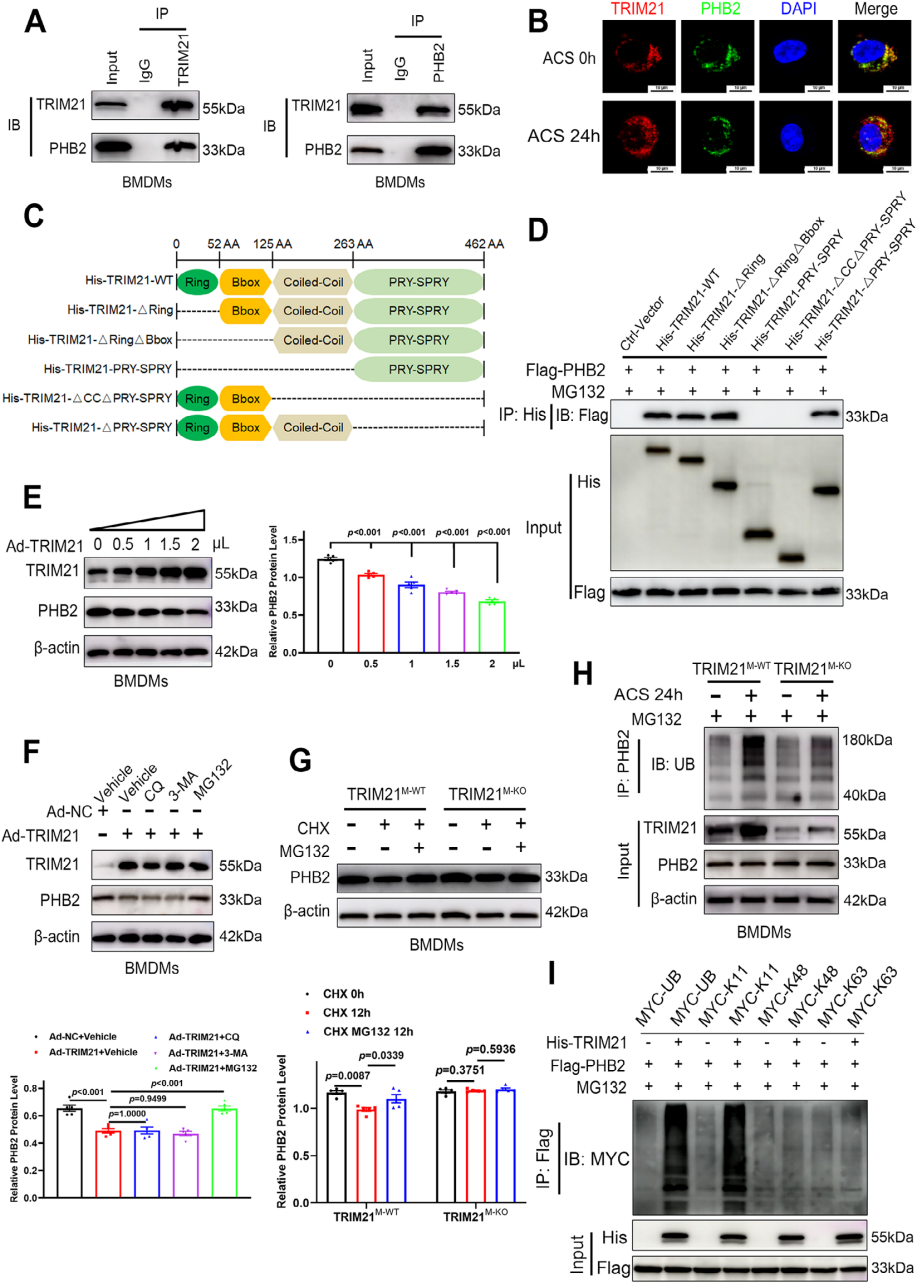
TRIM21 could bind to PHB2 via its coiled‐coil domain and promoted K11‐linked ubiquitination‐dependent proteasomal degradation. (A) Co‐IP with anti‐TRIM21 (left) or anti‐PHB2 (right) in BMDMs (n = 5). (B) Co‐localization of TRIM21 (red) with PHB2 (green) in BMDMs. Cell nuclei were stained with DAPI (blue) (scale bar: 10 µm; *n* = 5). (C) Domain diagrams of TRIM21 WT and deletion mutants. (D) Co‐IP analysis of the interactions of Flag‐PHB2 with His‐TRIM21 (WT or mutants) in HEK293T cells (*n* = 5). (E) Representative Western blotting images (left panel) and quantification (right panel) of PHB2 levels in BMDMs transfected with gradient concentrations of Ad‐*Trim21* (*n* = 5). (F) Representative Western blotting images (upper panel) and quantification (lower panel) of PHB2 levels in Ad‐*Trim21*‐expressing BMDMs pretreated with chloroquine (CQ; 50 µm), 3‐methyladenine (3‐MA; 10 mm), and Z‐Leu‐Leu‐Leu‐al (MG132; 10 µm) for 12 h (*n* = 5). (G) Representative Western blotting images (upper panel) and quantification (lower panel) of PHB2 levels in BMDMs from *Trim21^M‐WT^* or *Trim21^M‐KO^* mice treated with cycloheximide (CHX; 100 µg/mL) or MG132 (10 µm) for 0 h or 12 h (*n* = 5). (H) Co‐IP results for PHB2 ubiquitination levels. BMDMs from *Trim21^M‐WT^* or *Trim21^M‐KO^* mice were treated with MG132 (10 µm) for 12 h and with ACS for 12 h (*n* = 5). (I) Co‐IP results for exogenous PHB2 ubiquitination levels. HEK293T cells were transfected with the control vector, His‐TRIM21(WT), Flag‐PHB2, and MYC‐ubiquitin (WT/K11/K48/K63) (*n* = 5). Data are expressed as mean ± SEM (E–G). Statistical analyses were conducted using ANOVA with Tukey HSD post hoc test (E, F) and Kruskal‐Wallis test with Dunn's post hoc test (G).

Endogenous TRIM21‐PHB2 interaction was confirmed in BMDMs via co‐IP and immunofluorescence (Figure 5A,B). To investigate which interacting domains of TRIM21 bind to PHB2, we constructed His‐tagged full‐length TRIM21 and its truncated mutants (Figure 5C). Co‐IP assays revealed that deletion of the coiled‐coil domain abolished TRIM21‐PHB2 binding (Figure 5D).

We subsequently measured TRIM21‐mediated regulation of PHB2 stability. *Trim21* overexpression dose‐dependently downregulated endogenous PHB2 levels in BMDMs (Figure 5E). Given that the E3 ubiquitin ligase activity of TRIM21 may promote substrate degradation through the ubiquitin‐proteasome pathway or autophagic‐lysosomal pathway [25], we pharmacologically inhibited these pathways. Treatment with the proteasome inhibitor MG132, but not with CQ or 3‐MA, rescued *Trim21* overexpression‐induced PHB2 depletion, confirming proteasomal degradation as the dominant mechanism (Figure 5F). To confirm this finding, BMDMs were treated with CHX to block protein synthesis, resulting in reduced PHB2 levels. This reduction was reversed by MG132, but this effect was not observed after *Trim21* knockout (Figure 5G). Furthermore, ACS stimulation upregulated ubiquitinated PHB2 in MG132‐treated BMDMs, an effect abolished after *Trim21* deletion (Figure 5H). Taken together, TRIM21 degrades PHB2 through the ubiquitin‐proteasome pathway.

Since K11‐, K48‐, and K63‐linked polyubiquitination trigger proteasomal degradation [26], we co‐transfected HEK293T cells with His‐TRIM21, Flag‐PHB2, and MYC‐tagged ubiquitin (WT/K11/K48/K63) to delineate the specificity of the ubiquitin linkage. TRIM21 selectively mediated PHB2 ubiquitination in the presence of WT or K11‐linked ubiquitin, but not in the presence of K48‐ or K63‐linked ubiquitin (Figure 5I). Overall, macrophage TRIM21 was found to bind to PHB2 via its coiled‐coil domain and promote K11‐linked ubiquitination‐dependent proteasomal degradation.

### 2.6 Macrophage Phb2 Deletion Reversed the Ameliorative Effects of Trim21 Deficiency in AP

**FIGURE 6:**
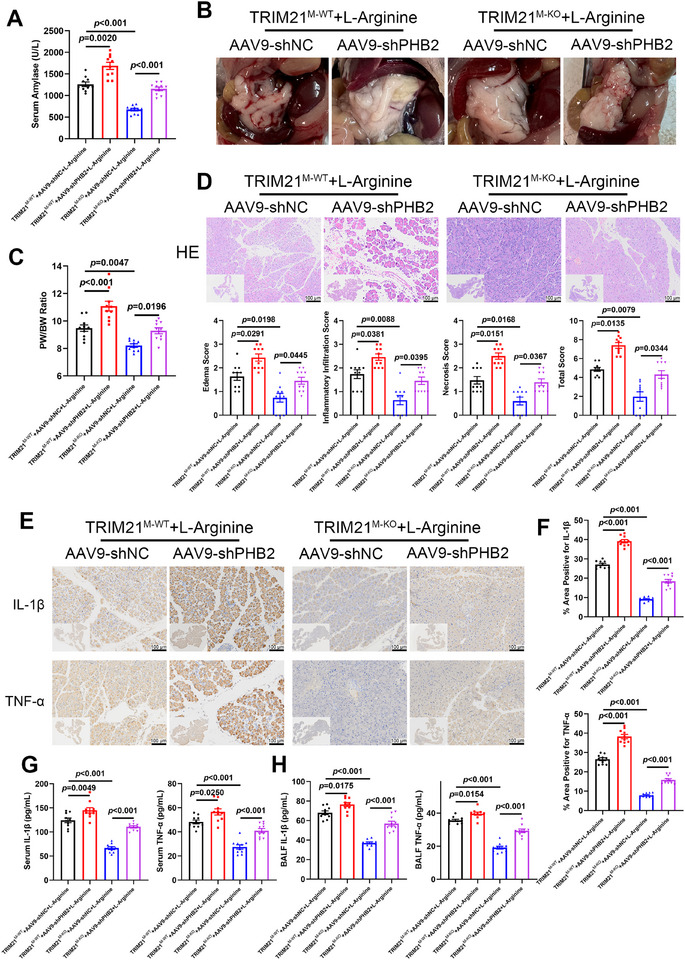
Macrophage *Phb2* deficiency reversed *Trim21* deficiency‐mediated improvement in AP. (A–H) *Trim21^M‐KO^* and *Trim21^M‐WT^* mice received AAV9‐sh*Phb2* or AAV9‐sh*NC* for 4 weeks, followed by 72 h of infusion of L‐arginine (*Trim21^M‐WT^* + AAV9‐sh*NC* + L‐arginine, *Trim21^M‐WT^* + AAV9‐sh*Phb2* + L‐arginine, *Trim21^M‐KO^* + AAV9‐sh*NC* + L‐arginine, *Trim21^M‐KO^* + AAV9‐sh*Phb2*+ L‐arginine, *n* = 10 per group). (A) Serum amylase levels. (B) Gross morphology of the pancreas. (C) Pancreas‐to‐body weight ratio. (D) H&E staining (upper panel) and histopathological scores (lower panel) of the pancreas. Representative images (E) and quantification (F) of IHC staining for IL‐1β and TNF‐α in the pancreatic tissue. (G, H) Serum and BALF levels of IL‐1β and TNF‐α. Data are expressed as mean ± SEM (A, C, D, F–H), and statistical analyses were conducted using Welch's ANOVA with Games‐Howell post hoc test (A, F), ANOVA with Tukey HSD post hoc test (C, G, H), and Kruskal‐Wallis test with Dunn's post hoc test (D).

To investigate whether macrophage TRIM21 exacerbates the progression of AP by regulating PHB2, *Trim21^M‐WT^* and *Trim21^M‐KO^* mice received tail vein injections of adeno‐associated virus of *Phb2* shRNA (AAV‐sh*Phb2*) four weeks before L‐arginine‐induced AP modeling, enabling macrophage‐specific *Phb2* knockdown. *Phb2* knockdown elevated serum amylase levels in AP *Trim21^M‐WT^* mice and abrogated suppression in AP *Trim21^M‐KO^* mice (Figure 6A). Compared to AP *Trim21^M‐WT^* controls, pancreatic edema and pancreas‐to‐body weight ratios were significantly increased in *Phb2*‐deficient counterparts (Figure 6B,C). Histopathological scoring revealed that *Trim21* knockdown ameliorated pancreatic injury, including interstitial edema, inflammatory infiltration, and acinar necrosis, but *Phb2* knockdown reversed this protective effect (Figure 6D). Combined IHC and ELISA analyses confirmed that *Phb2* knockdown in AP *Trim21^M‐KO^* mice negated the anti‐inflammatory benefits of *Trim21* deficiency in macrophages, evidenced by increased protein levels of IL‐1β and TNF‐α in the pancreas, serum, and BALF (Figure 6E–H). In vitro, *Phb2* knockdown upregulated pro‐inflammatory markers (iNOS, NLRP3, pro‐caspase‐1, ASC, pro‐IL‐1β, and TNF‐α) with reduced Arg‐1 levels observed in ACS‐treated BMDMs (Figure S4A,B). *Trim21* deletion effectively downregulated these pro‐inflammatory mediators, whereas *Phb2* knockdown restored their expression (Figure S6A,B). These results collectively suggest that macrophage TRIM21‐mediated post‐translational regulation of PHB2 intensifies the inflammatory response and accelerates the progression of AP.

### 2.7 Therapeutic Suppression of TRIM21 Prevented the Progression of AP and Suppressed the Inflammatory Response

**FIGURE 7:**
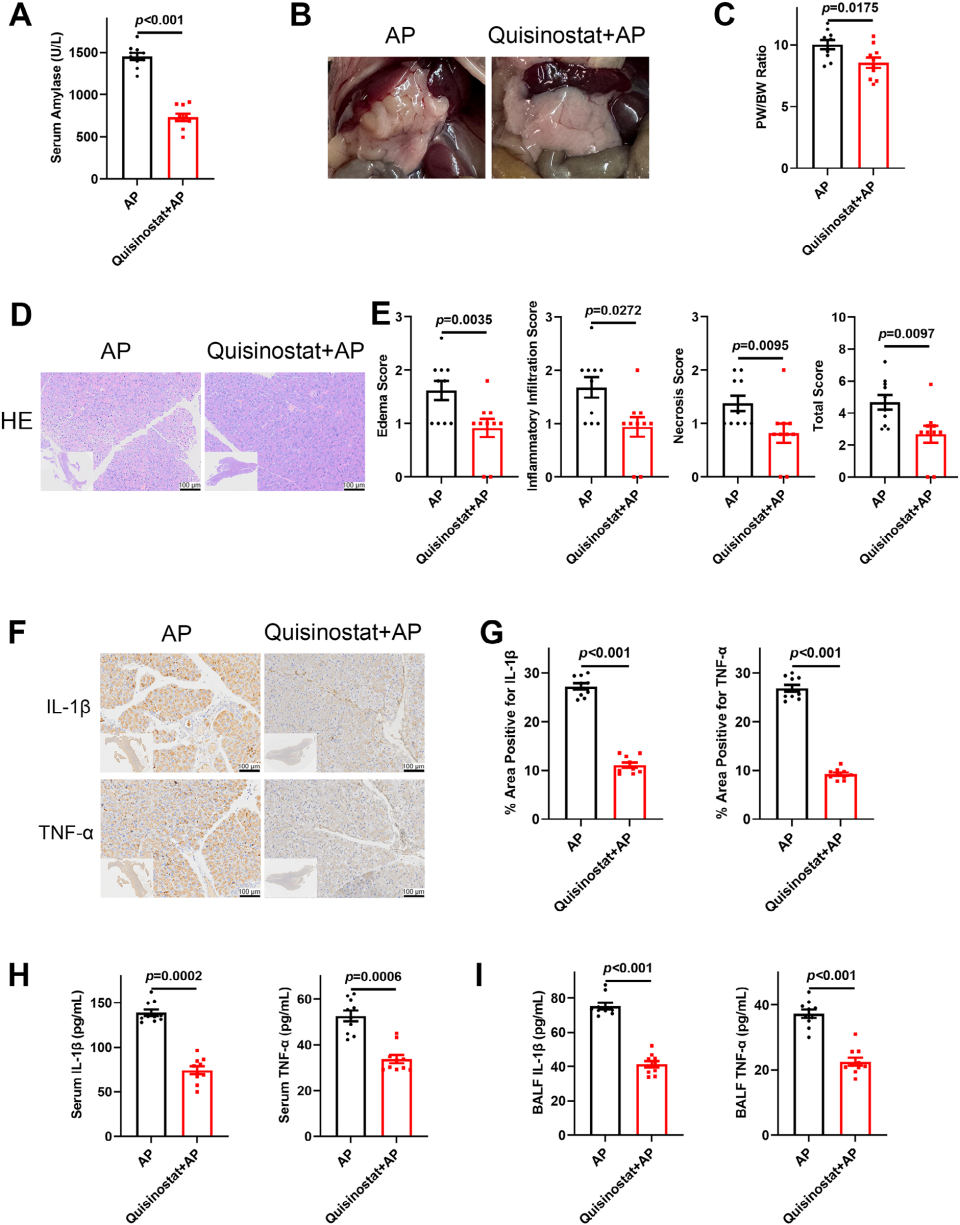
TRIM21 suppression mitigated L‐arginine‐induced development of AP. (A–I) *Trim21^M‐WT^* mice pretreated with vehicle or quisinostat intraperitoneally received L‐arginine for 72 h (AP, quisinostat + AP; *n* = 10 per group). (A) Serum amylase levels. (B) Gross morphology of the pancreas. (C) Pancreas‐to‐body weight ratio. H&E staining (D) and histopathological scores (E) of the pancreas. Representative images (F) and quantification (G) of IHC staining for IL‐1β and TNF‐α in pancreatic tissue. (H, I) IL‐1β and TNF‐α levels in serum samples and BALF. Data are expressed as mean ± SEM (A, C, E, G–I), and statistical analyses were conducted using Student's t‐test (A, C, IL‐1β in G, TNF‐α in I), Mann‐Whitney U test (E, H, IL‐1β in I), and Welch's t‐test (TNF‐α in G).

Our study revealed the therapeutic potential of quisinostat, a TRIM21 inhibitor [27], in preventing the progression of AP. In L‐arginine‐induced AP, pretreatment with quisinostat significantly reduced serum amylase levels (Figure 7A). Quisinostat‐treated mice exhibited attenuated pancreatic edema and decreased pancreas‐to‐body weight ratios after L‐arginine challenge (Figure 7B,C). H&E staining also confirmed the therapeutic effects, revealing reduced pancreatic edema, diminished inflammatory cell infiltration, and limited acinar necrosis in quisinostat‐treated mice (Figure 7D,E). Notably, quisinostat abrogated the pro‐inflammatory effects in L‐arginine‐induced AP. IHC and ELISA revealed that quisinostat downregulated IL‐1β and TNF‐α levels in the pancreas, serum, and BALF, suggesting that quisinostat suppresses the inflammatory cascade (Figure 7F–I). To investigate whether the therapeutic effect of quisinostat is mediated by the restoration of mitophagic flux, we examined its impact on key mitophagy markers in ACS‐stimulated BMDMs and found that its treatment restored the levels of PINK1, Parkin, and LC3B‐II and reduced p62 accumulation, indicating enhanced mitophagic flux (Figure S7A). To further substantiate TRIM21 as the key target of quisinostat, *Trim21^M‐KO^* and *Trim21^M‐WT^* mice received an intraperitoneal injection of quisinostat prior to L‐arginine‐induced AP modeling. H&E staining revealed that genetic deletion of *Trim21* ameliorated pancreatic injury. Importantly, quisinostat provided no additional protective effect in *Trim21^M‐KO^* mice beyond that conferred by the knockout alone (Figure S7B). This genetic evidence establishes that the therapeutic action of quisinostat in AP is primarily mediated by the inhibition of macrophage TRIM21. Thus, pharmacological inhibition of TRIM21 alleviates the progression of AP, highlighting TRIM21 as a novel therapeutic target and quisinostat as a promising therapeutic candidate for AP.

## 3 Discussion

**FIGURE 8:**
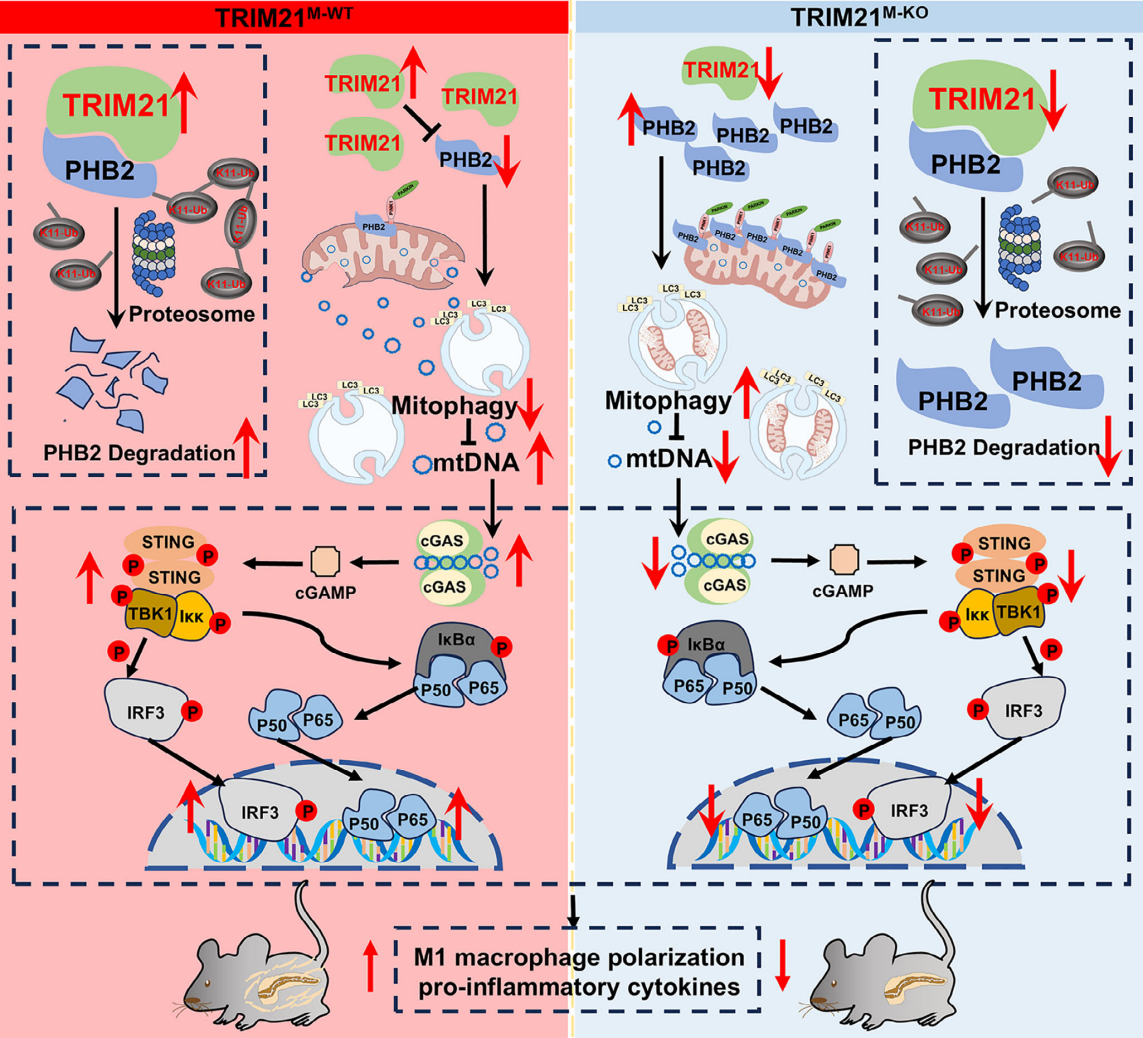
Proposed working model of TRIM21. Macrophage‐specific TRIM21 upregulation exacerbates the development of AP. Mechanistically, macrophage TRIM21 facilitates PHB2 degradation through ubiquitin‐proteasome processing, leading to cytosolic mtDNA accumulation via impaired PHB2‐mediated mitophagy. This mtDNA homeostasis disruption triggers cGAS‐STING axis activation, thereby exacerbating inflammatory cascades during AP progression.

AP is a life‐threatening inflammatory disorder progressing from local inflammation to a systemic disease, with 30% mortality observed in severe cases developing SIRS [2]. The pathological progression of AP depends on uncontrolled macrophage‐mediated inflammation, a hallmark feature that can even lead to MODS and death [28]. In this study, we found that macrophage‐specific *Trim21* ablation or pharmacological inhibition of Trim21 significantly mitigated the progression of AP. Conversely, TRIM21 activation intensified both local histopathological damage and the systemic inflammatory cascade. Mechanistically, macrophage TRIM21 facilitated PHB2 degradation through ubiquitin‐proteasome processing, leading to cytosolic mtDNA accumulation via impaired PHB2‐mediated mitophagy. Disruption of mtDNA homeostasis activates the cGAS‐STING axis, thereby exacerbating inflammatory cascades during the progression of AP (Figure 8). These mechanistic insights collectively suggest TRIM21 inhibition in macrophages as a viable therapeutic approach for AP, which acts by restoring PHB2 homeostasis.

Inflammatory cascades initiated by acinar cell injury represent a pivotal pathological hallmark of AP [3]. Macrophages, constituting the dominant immune infiltrate in early AP, amplify inflammatory dysregulation through NLRP3 inflammasome‐mediated pro‐inflammatory polarization [7, 29]. They lead to the excessive release of pro‐inflammatory mediators, such as IL‐1β and TNF‐α, that exacerbate local pancreatic damage and promote progression to SIRS and MODS [8]. Elevated TRIM21 expression was observed via immunofluorescence staining in AP mice, showing notable co‐localization with macrophages. Furthermore, TRIM21 levels in circulating monocytes were found to be significantly increased in both AP patients and AP mice relative to their corresponding controls, a finding reported here for the first time. The concurrent upregulation of TRIM21 in both pancreatic‐infiltrating macrophages and circulating monocytes suggests that its induction may occur through both local and systemic pathways. Under physiological conditions, classical monocyte activation is generally a tissue‐localized event. However, in severe systemic inflammatory diseases such as AP, substantial amounts of DAMPs, cytokines (e.g., IL‐1β, TNF‐α), and other inflammatory mediators are released into the circulation, establishing a pro‐inflammatory systemic milieu [30]. In this setting, circulating monocytes can undergo functional reprogramming in response to systemic inflammatory stimuli [31, 32]. This mechanism is not without precedent. For example, in patients with sepsis—another condition characterized by a systemic cytokine storm—peripheral blood mononuclear cells (PBMCs) exhibit significant alterations in the expression of various inflammatory regulators, often correlating with disease severity [33]. Although the specific upregulation of TRIM21 in circulating monocytes during AP has not been extensively characterized until now, our data provide direct evidence supporting this phenomenon. This finding enhances our understanding of the systemic dimension of AP and underscores the role of TRIM21 as a central inflammatory node that can be regulated both locally and remotely. Having established the upregulation of TRIM21 in AP, we next sought to determine its functional role in disease pathogenesis through genetic and pharmacological interventions. In our study, macrophage‐specific *Trim21* deficiency substantially attenuated pro‐inflammatory macrophage polarization, suppressed NLRP3 inflammasome activation, and inhibited the overproduction of inflammatory cytokines, such as IL‐1β and TNF‐α. Conversely, pharmacological activation of TRIM21 using vilazodone exacerbated the inflammatory cascade and disease severity. Collectively, these results suggest that macrophage TRIM21 orchestrates inflammatory dysregulation through hierarchical control of inflammatory signaling networks, thereby functioning as a central regulator of AP.

Pathological stress conditions induce cytosolic mtDNA accumulation, critically activating the cGAS‐STING axis, which is a central mechanism driving pro‐inflammatory cascades in AP [29, 34, 35]. This axis coordinates the phenotypic reprogramming of macrophages and pro‐inflammatory cytokine secretion by activating downstream effectors [36, 37]. Combined with the results of our proteomic profiling, these findings suggest that macrophage TRIM21 may enhance the inflammatory response by modulating the protein levels of PHB2 and the cGAS‐STING signaling axis in AP. PHB2 has recently been identified as a mitophagy‐regulating protein acting through two mechanisms. It functions not only as an inner mitochondrial membrane autophagy receptor that recruits LC3B to mediate PINK1‐Parkin‐dependent mitophagy [17] but also stabilizes the intramitochondrial protease PARL to prevent the proteolytic cleavage of PGAM5. Intact PGAM5 maintains PINK1 stabilization on the outer mitochondrial membrane [18], thereby facilitating Parkin recruitment and mitophagy initiation. Mitophagy, as an essential quality control mechanism, selectively eliminates damaged mitochondria to maintain organellar homeostasis. Impaired mitophagy triggers mtDNA leakage into the cytosol. As a central activator of the cGAS‐STING signaling pathway, cytosolic mtDNA engages this DNA‐sensing cascade to drive macrophage polarization toward a pro‐inflammatory phenotype, thereby exacerbating tissue inflammation, promoting cytokine storm, and amplifying systemic inflammatory responses [19, 38]. Our study revealed that in AP, macrophage TRIM21 interacts with PHB2 and mediates its degradation via the ubiquitin‐proteasome pathway. Critically, TRIM21 deficiency enhanced the protein expression of PHB2, promotes mitophagic flux, prevents cytosolic mtDNA accumulation, and suppresses the STING pathway. In contrast, TRIM21 overexpression produces diametrically opposite effects. Rescue experiments definitively indicated that TRIM21‐mediated aggravation of AP requires ubiquitin‐dependent degradation of PHB2. These findings align with previous studies establishing PHB2 as a molecular gatekeeper of the STING signaling cascade through its role in maintaining mitochondrial homeostasis and restricting mtDNA release [18, 19, 23]. In sum, macrophage TRIM21 degrades PHB2 via the ubiquitin‐proteasome pathway, which upregulates cytosolic mtDNA by inhibiting PHB2‐mediated mitophagy, thereby activating the cGAS‐STING signaling pathway to drive inflammatory cascades in AP. Importantly, our findings are not limited to a single experimental model. The protective effect of macrophage *Trim21* deletion and the associated reversal of mitophagy impairment and cGAS‐STING activation were consistently observed in both L‐arginine‐ and caerulein‐induced AP models. As these two models initiate pancreatitis through distinct mechanisms (direct mitochondrial toxicity versus supramaximal secretory stimulation), the conservation of the TRIM21‐PHB2 axis across them underscores its role as a central regulator of innate inflammatory responses in AP, rather than a model‐specific artifact. This broad relevance strengthens the potential translational significance of targeting this pathway.

TRIM21, a multi‐domain E3 protein ligase, exhibits domain‐specific regulation of ubiquitin‐dependent proteolysis. Current evidence suggests that its RING domain mediates antiviral defense [39], while the coiled‐coil and PRY/SPRY domains coordinate diverse molecular interactions governing inflammatory responses [40], autophagy [41], and tumor cell proliferation [27]. Our Co‐IP analyses revealed binding between the coiled‐coil domain of TRIM21 and PHB2, triggering its subsequent proteasomal degradation. TRIM21 can catalyze K11‐linked [41, 42], K48‐linked [43], and K63‐linked [44] ubiquitin modifications on specific substrates. Ubiquitination assays employing MYC‐tagged ubiquitin (WT/K11/K48/K63) showed that TRIM21 exclusively promotes PHB2 degradation via K11‐linked polyubiquitination. Previous studies indicated that quisinostat can suppress endogenous TRIM21 [27]. In addition, the histone deacetylase inhibitor quisinostat was found to target caspase‐1‐mediated and NF‐κB‐mediated inflammation [45, 46], substantiating its anti‐inflammatory properties. To clarify the relevant mechanism of action in AP, we performed a critical genetic validation. We found that the therapeutic effect of quisinostat was abolished in macrophage‐specific *Trim21* knockout mice, which were already protected from AP. The absence of any additive benefit in *Trim21^M‐KO^* mice indicates that TRIM21 inhibition is a major pathway through which quisinostat exerts protection in our AP model. Consistently, quisinostat treatment attenuated pro‐inflammatory macrophage polarization, reduced IL‐1β and TNF‐α production, and diminished inflammatory cell infiltration. Crucially, this multipronged suppression ameliorated pancreatic edema and necrosis, interrupted systemic inflammatory cascades to confer multi‐organ protection, and retarded the pathological progression of AP—effects largely attributable to its action on the macrophage TRIM21‐PHB2 axis in this context.

It is also important to acknowledge the limitations of our study. (1) The lack of rescue experiments using pharmacological inhibitors or agonists of mitophagy to further cement the central role of TRIM21 in AP by mediating mitophagy; (2) Vilazodone and quisinostat are not exclusively specific to TRIM21. While we confirmed that their main effects in AP are mediated by targeting TRIM21, whether additional molecular mechanisms contribute to their roles in AP remains to be further explored; (3) Although macrophages are the primary cells involved in pancreatic inflammatory responses and the secretion of pro‐inflammatory cytokines [4], prior literature indicates that acinar cells may also contribute partially to the secretion of pro‐inflammatory cytokines [47], which has not been confirmed in our study.

## 4 Conclusions

Our study elucidated the pivotal role of macrophage TRIM21 in the pathogenesis of AP. Increased expression of macrophage TRIM21 directly exacerbates both local inflammation and systemic inflammation in AP. This pathogenic cascade was ameliorated by macrophage‐specific *Trim21* ablation, which substantially protected the pancreas by suppressing pro‐inflammatory signaling. Specifically, macrophage‐derived TRIM21 drives the progression of AP via ubiquitin‐proteasome‐mediated degradation of PHB2, leading to impaired PHB2‐mediated mitophagy. Therefore, accumulation of cytosolic mtDNA hyperactivates the cGAS‐STING signaling axis, thereby amplifying inflammatory cascades. The TRIM21‐PHB2‐mtDNA‐STING regulatory axis provides a mechanistic framework for macrophage‐based treatment of AP, wherein both AAV‐delivered sh*TRIM21* vectors and pharmacological inhibition of TRIM21 prevent inflammation‐driven pancreatic damage, SIRS progression, MODS development, and mortality in AP.

## 5 Materials and Methods

### 5.1 Human Subjects

This study consecutively enrolled a total of 130 participants, comprising 101 patients with AP admitted within 24 h at the Emergency Department of the Second Affiliated Hospital of Anhui Medical University (May 2021 to October 2021) and 29 age‐matched healthy volunteers recruited from the institutional health screening center who served as controls. Diagnosis of AP and its severity were defined following the revised Atlanta classification [48]. The exclusion criteria were as follows: (1) age <18 or >65 years; (2) traumatic or post‐endoscopic retrograde cholangiopancreatography (ERCP)‐induced pancreatitis; (3) pre‐existing malignancy or other active infectious/contagious diseases; (4) pregnancy; (5) symptom onset‐to‐admission interval >48 h; and (6) incomplete medical records. Peripheral blood samples were collected within 24 h after admission. Baseline characteristics of the healthy volunteers are listed in Table S1. Clinical, demographic, key comorbidities, and routine laboratory data were collected from the patients' electronic medical records (Table S1). The study protocol was approved by the institutional ethics committee (Approval No. YX2021‐046 (F1)), and the study was conducted following the Declaration of Helsinki. Informed consent was provided by all participants before enrollment.

### 5.2 Murine AP Models and Treatment

All animal experiments strictly adhered to the ARRIVE guidelines (https://arriveguidelines.org) and were approved by the Animal Ethics Committee of Shandong University (Approval No. DWLL‐202500249). To establish macrophage‐specific *Trim21* knockout mice (*Trim21* ^M‐KO^: *Trim21* ^flox/flox^ *Lyz2‐Cre* ^+^), *Trim21* ^flox/flox^ mice were crossed with *Lyz2‐Cre* ^+/−^ mice (View Solid Biotechnology Inc., Beijing, China). Littermate controls (*Trim21* ^M‐WT^: *Trim21* ^flox/flox^ *Lyz2‐Cre* ^−^) were bred in parallel. All animals were housed under a specific pathogen‐free environment with ad libitum access to irradiated feed and autoclaved water. Environmental enrichment included nesting materials and staggered light‐dark cycles. The murine model of AP was constructed as described previously [49, 50]. Briefly, mice (6–8 weeks old) were fasted for 12 h with ad libitum access to water. AP was induced by two intraperitoneal injections of L‐arginine (4 g/kg, Sigma–Aldrich, St. Louis, MO, USA) at 1‐h intervals. Samples were collected 72 h after the first injection of L‐arginine. To assess potential sex‐specific effects, the L‐arginine‐induced AP model was established in age‐matched male and female mice as described above. For the caerulein‐induced AP model, mice received seven intraperitoneal injections of caerulein (50 µg/kg; MedChemExpress, Monmouth Junction, NJ, USA) at 1‐h intervals. Samples were collected 12 h after the first caerulein injection. TRIM21 was activated using vilazodone (25 mg/kg; MedChemExpress) and downregulated using quisinostat (10 mg/kg; MedChemExpress). Both were administered intraperitoneally 24 h before the induction of AP, and subsequent doses were given every other day.

### 5.3 Adeno‐Associated Virus, Adenovirus, and Viral Delivery Protocol

The macrophage‐specific *Phb2* knockdown group received the pAV‐*Lyz2*‐sh*Phb2*‐GFP vector (AAV9‐sh*Phb2*; 1 × 10^^12^ µg), while the control group was treated with the pAV‐*Lyz2*‐sh*NC*‐GFP vector (AAV9‐sh*NC*; 1 × 10^^12^ µg). The sh*Phb2* sequence was 5′‐CCAGAATATCTCCAAGACGAT‐3′. To establish *Trim21* overexpression, BMDMs were infected with Ad‐*Trim21* for 12 h, with Ad‐*NC* as a control. All viral preparations were commercially synthesized by Weizhen Biotechnology (Shandong, China).

### 5.4 Cell Isolation, Differentiation, and Treatment

Human/mouse PBMCs were isolated from whole blood using a species‐specific PBMCs separation kit (Solarbio, Beijing, China). Primary pancreatic acinar cells were isolated as described previously [51]. Briefly, the pancreatic tissue was minced in ice‐cold HBSS, followed by enzymatic digestion and subsequent purification. The isolated cells were cultured in suspension using DMEM supplemented with 1% FBS and 0.25 mg/mL SBTI. ACS were collected at designated time‐points: ACS 0 h (30 min post‐plating) and ACS 24 h (after 24‐h culture). BMDMs were isolated and cultured using a previously established protocol [13]. Briefly, BMDMs were extracted from the femurs and tibias and differentiated for 7 days in DMEM containing 20 ng/mL macrophage colony‐stimulating factor, 10% FBS, and 1% penicillin/streptomycin. For stimulation, BMDMs were treated with ACS for 12 h.

### 5.5 Transfection of Plasmids and siRNA

Transient transfection of siRNA or plasmids into BMDMs or HEK293T cells was conducted using Lipofectamine 3000 reagent (Invitrogen, Carlsbad, CA, USA), following the manufacturer's protocol. The targeting sequence for mouse *Phb2* siRNA was 5′‐CCAGAATATCTCCAAGACGAT‐3′.

### 5.6 Western Blotting

Cellular proteins were extracted using RIPA lysis buffer (Beyotime, Shanghai, China) containing 1× protease/phosphatase inhibitors. Details of the primary antibodies are provided in Table S4. Signal intensities were normalized to GAPDH/β‐actin levels.

### 5.7 Nucleic Acid Extraction and Quantitative Real‐Time PCR (qRT‐PCR) Analysis

Total RNA was isolated using TRIzol reagent (TIANGEN, Beijing, China), followed by reverse transcription with the HiScript III RT SuperMix kit (Vazyme, Nanjing, China), following the manufacturer's protocol. Cytoplasmic mtDNA release was detected as described previously [52]. Briefly, cytoplasmic DNA was extracted from cultured BMDMs using a mitochondria isolation kit (Thermo Fisher Scientific, MA, USA), purified to remove residual organellar DNA with the Monarch PCR & DNA Cleanup Kit (New England Biolabs, Ipswich, MA, USA), and amplified with gene‐specific primers. The results were normalized to 18S ribosomal RNA. Primers sequences are provided in Table S4.

### 5.8 Histological, IHC, and Immunofluorescent Staining

Murine pancreatic tissue fixed in 4% paraformaldehyde was stained following the manufacturer's protocol. The details of primary antibodies are shown in Table S4. H&E‐stained pancreas was graded from 0 to 3 as described previously [53], with five random fields analyzed blindly for each mouse. For quadruple fluorescence staining, we employed a triple antibody labeling kit (Afantibody, Hunan, China). Images were analyzed using an LSM 710 laser confocal microscope (Carl Zeiss AG, Jena, Germany).

### 5.9 ELISA

Protein levels were quantified by commercial ELISA kits following the manufacturers' specifications: TRIM21 (Ruixin Biotech, Fujian, China), IL‐1β, TNF‐α (MULTI SCIENCES, Zhejiang, China), and amylase (Nanjing Jianchen, Nanjing, China).

### 5.10 LC‐MS/MS, Proteomic Sequencing, and Bioinformatics Analysis

The proteomic analysis and LC‐MS/MS services were provided by Sinotech Genomics (Shanghai, China). The LC‐MS/MS analysis was performed using data‐dependent acquisition (DDA) mode on a Q Exactive HF‐X mass spectrometer. Full scan MS spectra (*m/z* 350–1500) were acquired, followed by higher‐energy collisional dissociation (HCD) fragmentation of the top 40 most intense precursor ions. For proteomic analysis, DEPs were defined as those with *p* < 0.05 and a fold change (FC) > 1.5 or < 1/1.5.

### 5.11 Collection and Analysis of GEO Data

To identify a robust and evolutionarily conserved TRIM gene in AP, we analyzed gene expression datasets from public GEO repositories. Our analysis incorporated mouse pancreatic tissue (GSE109227), mouse peripheral blood (GSE244335; samples GSM7813718‐GSM7813723), and human peripheral blood (GSE194331); no relevant dataset for human pancreatic tissue was available. We identified differentially expressed TRIM genes in each dataset using the “limma” R package. Subsequently, a Venn diagram analysis was applied to integrate these findings, which pinpointed the overlapping TRIM gene as a stable candidate.

### 5.12 Statistical Analysis

Statistical analysis and visualization were conducted using R software (version 4.4.2; R Foundation for Statistical Computing, Vienna, Austria) and GraphPad Prism 9 (GraphPad Software, San Diego, CA, USA). The normality of data was assessed using the Shapiro‐Wilk test. The homogeneity of variances was evaluated with Levene's test. Normally distributed data are expressed as mean ± standard deviation (SD). Such data were analyzed using the following analytical approaches: Student's t‐test to compare two groups when variances were equal; Welch's t‐test for two‐group comparisons when variances were unequal; One‐way ANOVA for multi‐group comparisons in the presence of homogeneous variances; Welch's ANOVA for multi‐group comparisons with heterogeneous variances. Non‐normally distributed continuous variables are reported as median and interquartile range (IQR). We employed the Mann‐Whitney U test for two‐group comparisons and the Kruskal‐Wallis test for multi‐group analyses. Categorical data were analyzed using Pearson's chi‐square test, Yates' corrected chi‐square test, or Fisher's exact test. We selected the criteria based on sample sizes and expected cell frequencies. All experiments were conducted on at least three biological replicates, and all statistical tests were two‐tailed. *p* < 0.05 was considered statistically significant.

## Author Contributions

Y.X., Y.S., X.Z., F.X., and H.L. designed the research. Y.X., X.Z., K.S., C.Y., and Z.W. performed the experiments. Y.X., Y.S., K.S., and Z.W. analyzed data. Y.X., Y.S., C.Y., Z.W., F.X., and H.L. conceived the project, reviewed the data, and wrote the manuscript. All authors contributed to the article and approved the submitted version.

## Funding

This work was supported by the National Natural Science Foundation of China (82302429), Natural Science Foundation of Shandong Province (ZR2025MS1355, ZR2023QH262), Anhui Provincial Health Research Project (AHWJ2023A20119), and the Anhui Medical University NSFC Mentorship Program 2023 (2023GMFY02).

## Conflicts of Interest

The authors declare no conflicts of interest.

## Supporting information




**Supporting File 1**: advs74024‐sup‐0001‐SuppMat.docx.

**Supporting File 2**: advs74024‐sup‐0002‐TableS2.xlsx.

**Supporting File 3**: advs74024‐sup‐0003‐TableS3.xlsx.

## Data Availability

All original data used for this study are available from the corresponding authors upon reasonable request.
