# Supplementary material for: Macrophage TRIM21 Inhibition Ameliorates Murine Acute Pancreatitis via PHB2‐Mediated Mitochondrial Stabilization
Source: Adv Sci (Weinh). 2026 Jan 22;13(19):e17877. doi: 10.1002/advs.202517877 (PMC13045386; doi:10.1002/advs.202517877)
Supplement: Supplementary file 1 — Supporting File 1: advs74024‐sup‐0001‐SuppMat.docx. [file ADVS-13-e17877-s002.docx]

Supporting Information

**Macrophage TRIM21 Inhibition Ameliorates Murine Acute Pancreatitis via PHB2-Mediated Mitochondrial Stabilization**

*Yansong Xu^1, 2, 3, 4, 5, 6^, Yuansong Sun^6^, Xin Zhou^6^, Kai Song^6^, Chunlin Yin^6^, Zhaohua Wang^6^, Fei Xie^1, 2, 3, 4, 5 *^, He Li ^6^* *^*^*

Contents

[Supporting Information Figures 3](#_Toc217036983)

[Supporting Information Tables 13](#_Toc217036984)

# Supporting Information Figures

**
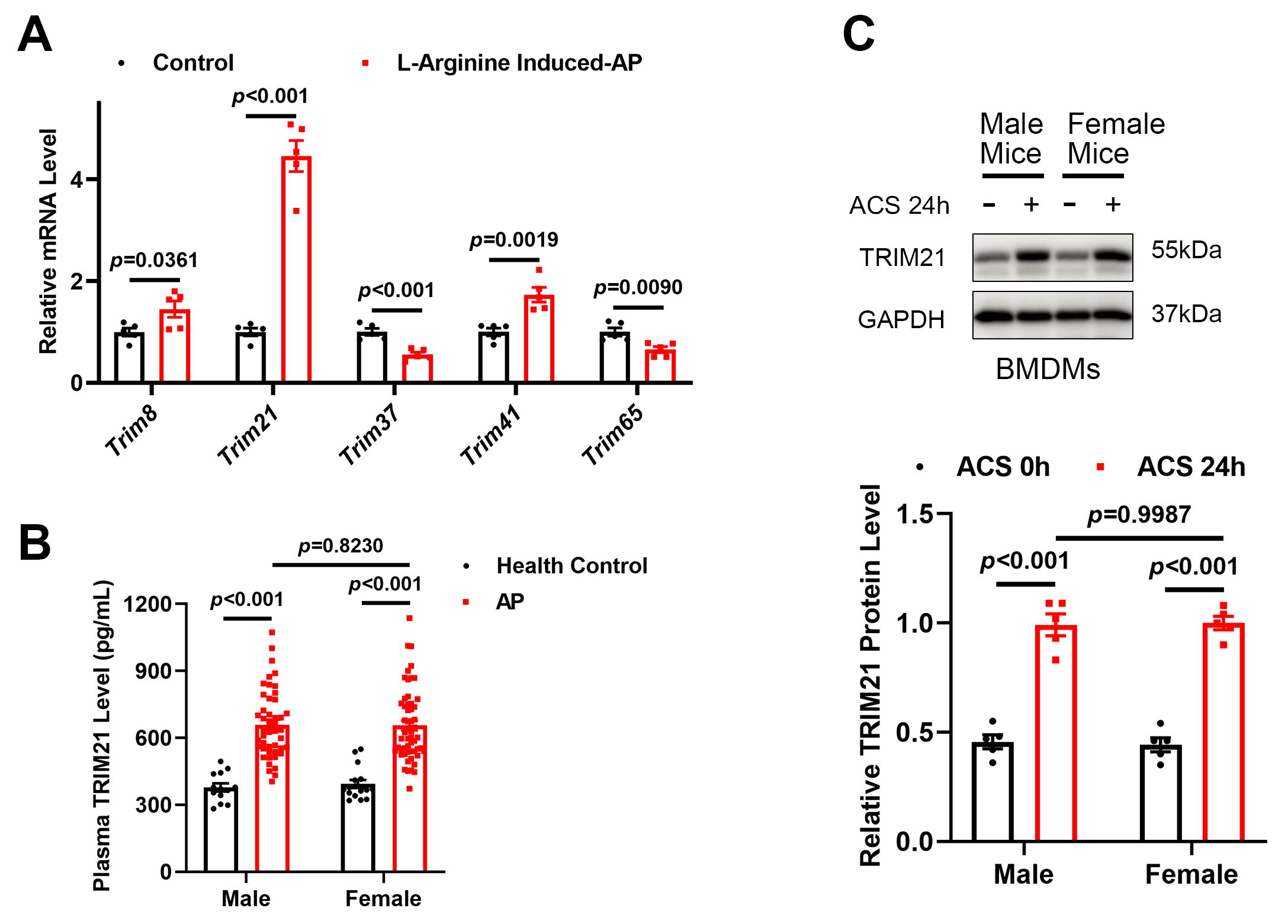
**

**Figures S1.** TRIM21 is upregulated in acute pancreatitis (AP) and shows no sex-specific expression differences. (A) RT-qPCR analysis of mRNA levels for TRIM genes (*Trim8*, *Trim21*, *Trim37*, *Trim41*, and *Trim65*) (n = 5). (B) The plasma levels of TRIM21 in healthy controls (13 males and 16 females) and patients with AP (46 males and 55 females) of both sexes. (C) Western blot analysis of TRIM21 protein levels in bone marrow-derived macrophages (BMDMs) from male and female mice stimulated with acinar cell supernatants (ACS) (n = 5). Data are expressed as mean ± SEM (A-C), and statistical analyses were conducted using Welch's t-test (*Trim21* in A), Student's t-test (indicators other than *Trim21* in A), Kruskal-Wallis test with Dunn's post hoc test (B), and ANOVA with Tukey HSD post hoc test (C).


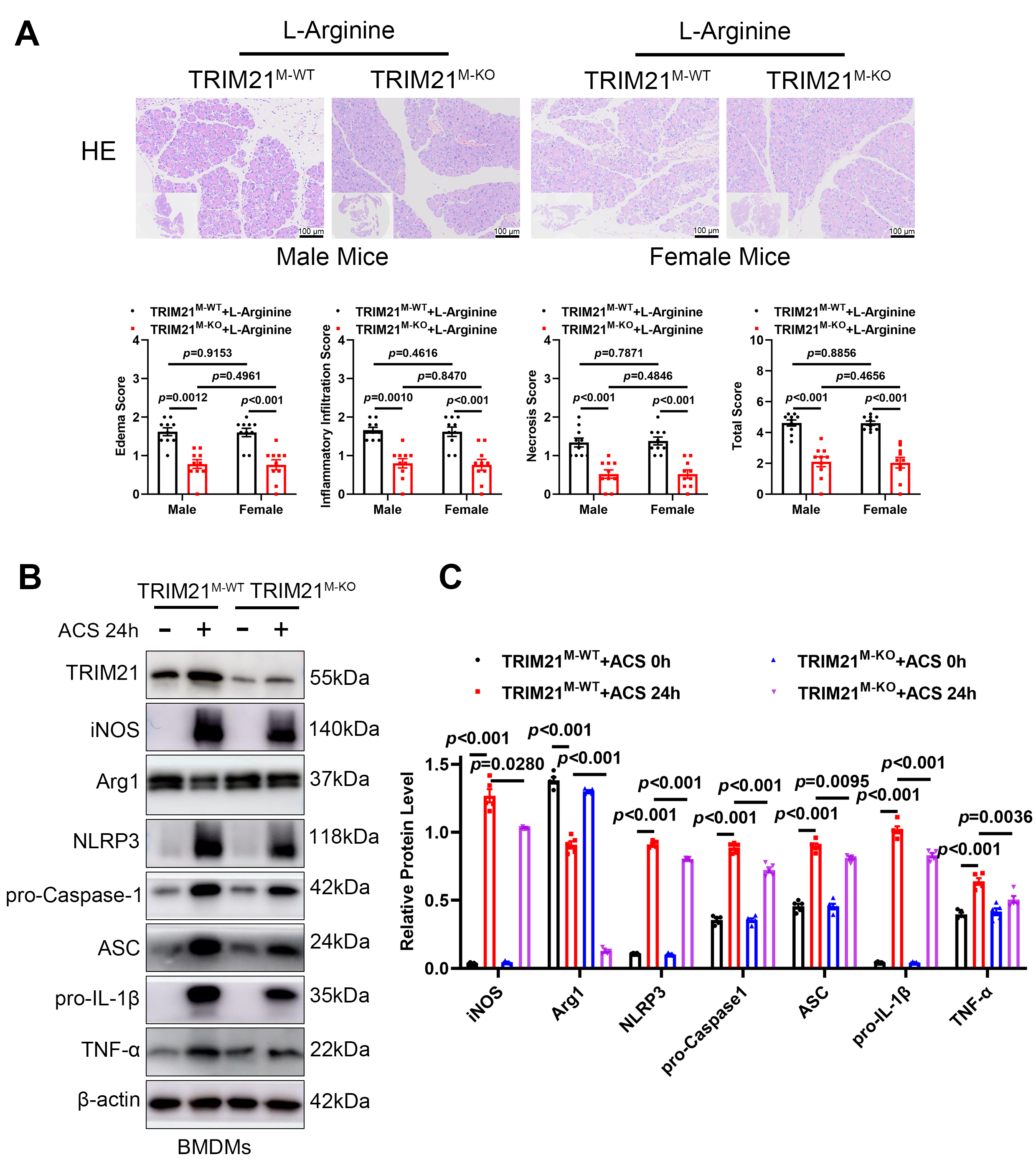


**Figure S2.** Effects of macrophage-specific *Trim21* knockout in L-arginine-induced AP and ACS-stimulated BMDMs. (A) Hematoxylin and eosin (H&E) staining (upper panel) and histopathological scoring (lower panel) of pancreatic tissue. *Trim21^M-KO^* and *Trim21^M-WT^* mice of both sexes intraperitoneally received L-arginine for 72 hours (n = 10 per group). (B and C) Representative Western blot images and quantiﬁcation of phenotype-specific proteins (iNOS and Arg1) and inflammatory proteins (NLRP3, pro-caspase-1, ASC, pro-IL-1β, TNF-α) in BMDMs from *Trim21^M-WT^* or *Trim21^M-KO^* mice after 12-hour ACS stimulation (n = 5). Data expressed as mean ± SEM (A, C). Statistical analyses were performed using Kruskal-Wallis test with Dunn's post hoc test (A), Welch's ANOVA followed by Games-Howell post hoc test (iNOS in C), and ANOVA followed by Tukey HSD post hoc test (indicators other than iNOS in C).


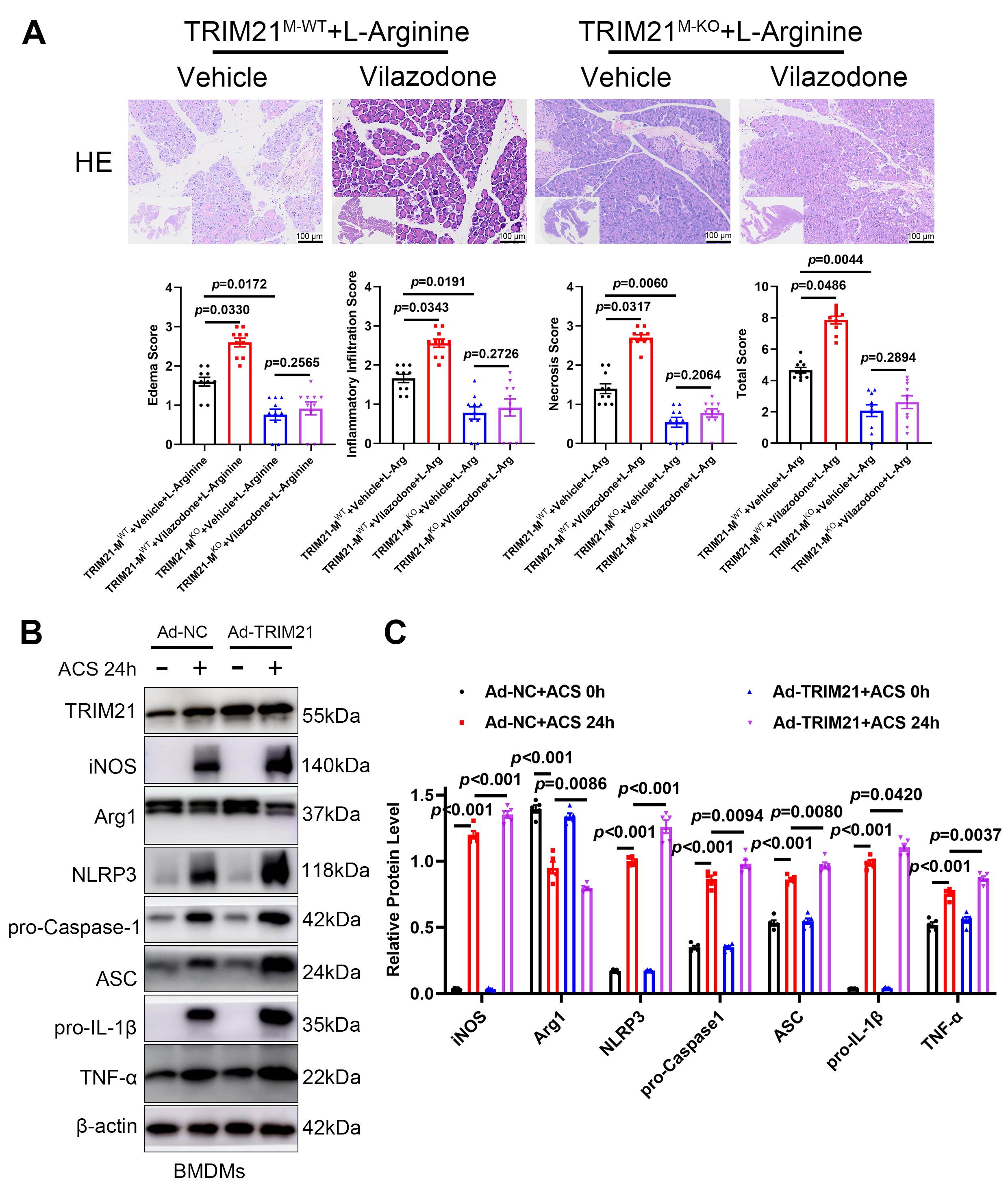


**Figure S3.** TRIM21-dependent exacerbation of AP by vilazodone and pro-inflammatory macrophage polarization by TRIM21 overexpression. (A) *Trim21^M-KO^* and *Trim21^M-WT^* mice pretreated with vehicle or vilazodone intraperitoneally received L-arginine for 72 hours (*Trim21^M-WT^* + Vehicle + L-arginine, *Trim21^M-WT^* + Vilazodone + L-arginine, *Trim21^M-KO^* + Vehicle + L-arginine, *Trim21^M-KO^* + Vilazodone + L-arginine; n = 10 per group). (A) H&E staining (upper panel) and histopathological scoring (lower panel) of pancreatic tissue. (B and C) Representative Western blot images and quantiﬁcation of phenotype-specific proteins (iNOS and Arg1) and inflammatory proteins (NLRP3, pro-caspase-1, ASC, pro-IL-1β, TNF-α) in BMDMs from *Trim21^M-WT^* mice transfected with an adenovirus carrying *Trim21* (Ad-*Trim21*) and then stimulated with ACS for 12 hours (n = 5). Data expressed as mean ± SEM (A, C). Statistical analyses were performed using Kruskal-Wallis test with Dunn's post hoc test (A), Welch's ANOVA followed by Games-Howell post hoc test (pro-IL-1β in C), and ANOVA followed by Tukey HSD post hoc test (indicators other than pro-IL-1β in C).


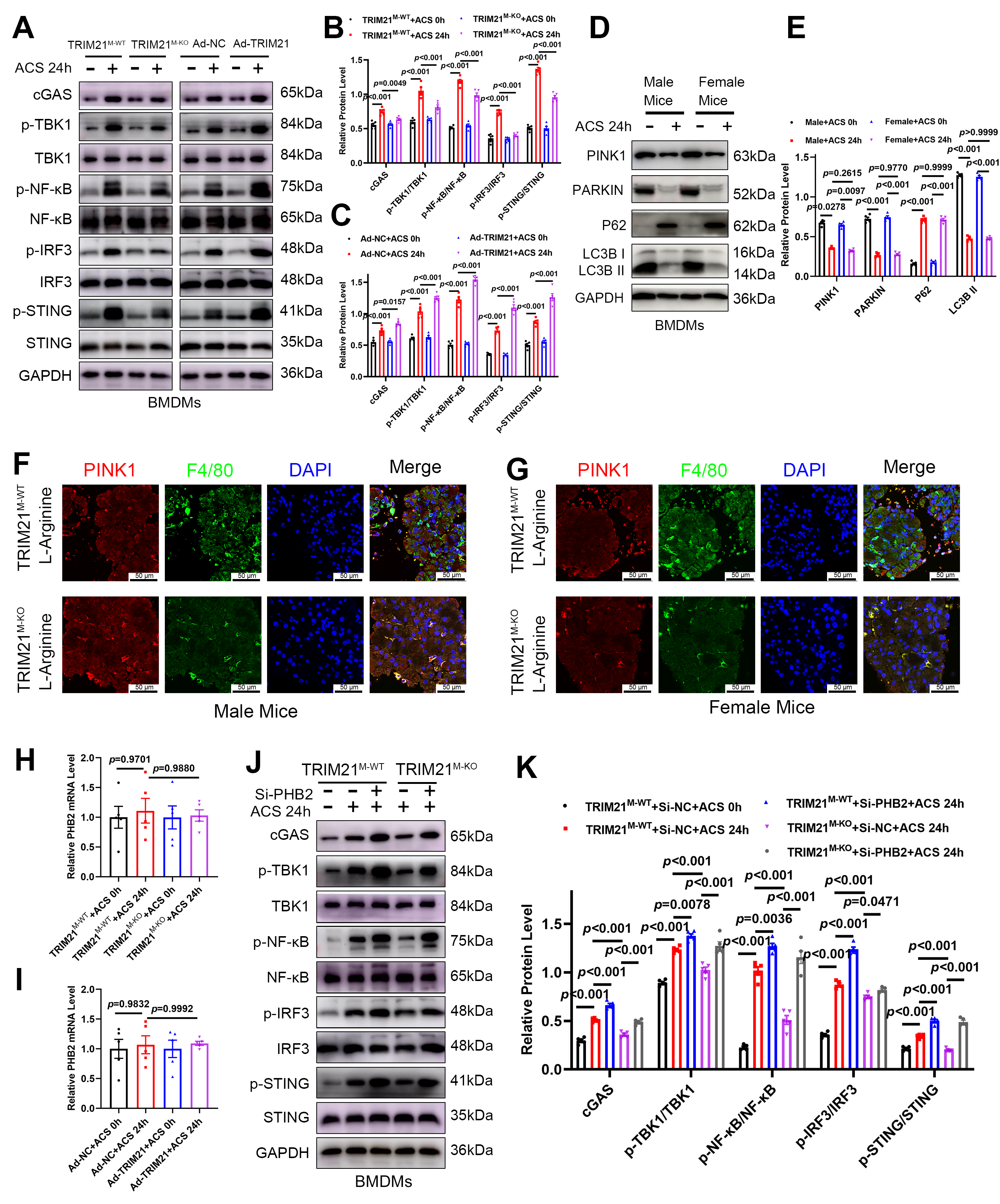


**Figure S4.** PHB2 mediates TRIM21-dependent regulation of mitophagy and cGAS-STING signaling in BMDMs. (A-C) Western blotting of the phosphorylation status (p-TBK1, p-NF-κB, p-IRF3, and p-STING) and total protein levels (cGAS, TBK1, NF-κB, IRF3, and STING) in BMDMs from *Trim21^M-WT^* and *Trim21^M-KO^* mice. *Trim21^M-WT^* mice were transfected with Ad-*Trim21* and then stimulated with ACS for 12 hours (n = 5). (D, E) Representative Western blot images (D) and quantiﬁcation (E) of mitophagy-associated protein levels (PINK1, Parkin, p62, and LC3B) in BMDMs from male and female mice stimulated with ACS (n = 5). (F, G) Representative co-immunofluorescence images of PINK1 (red) and the macrophage marker F4/80 (green) in pancreatic tissues from age-matched *Trim21^M-KO^* and *Trim21^M-WT^* mice of both sexes with L-arginine-induced AP. Cell nuclei were stained with DAPI (scale bar: 50 μm; n = 5). (H, I) RT-qPCR analysis of PHB2 mRNA levels in BMDMs from *Trim21^M-WT^* or *Trim21^M-KO^* mice (H), or *Trim21^M-WT^* mice transfected with Ad-*Trim21* (I) and then stimulated with ACS for 12 hours (n = 5). (J, K) Representative Western blot images (J) and quantiﬁcation (K) of phosphorylation status (p-TBK1, p-NF-κB, p-IRF3 and p-STING) and total protein levels (cGAS, TBK1, NF-κB, IRF3 and STING) in BMDMs from *Trim21^M-WT^* or *Trim21^M-KO^* mice following transfection with si-*Phb2* and subsequent 12-hour ACS treatment (n = 5). Data expressed as mean ± SEM (B, C, E, H, I, K). Statistical analyses were performed using ANOVA followed by Tukey HSD post hoc test (B, C, indicators other than PINK1 in E, H, I, K) and Kruskal-Wallis test with Dunn's post hoc test (PINK1 in E).


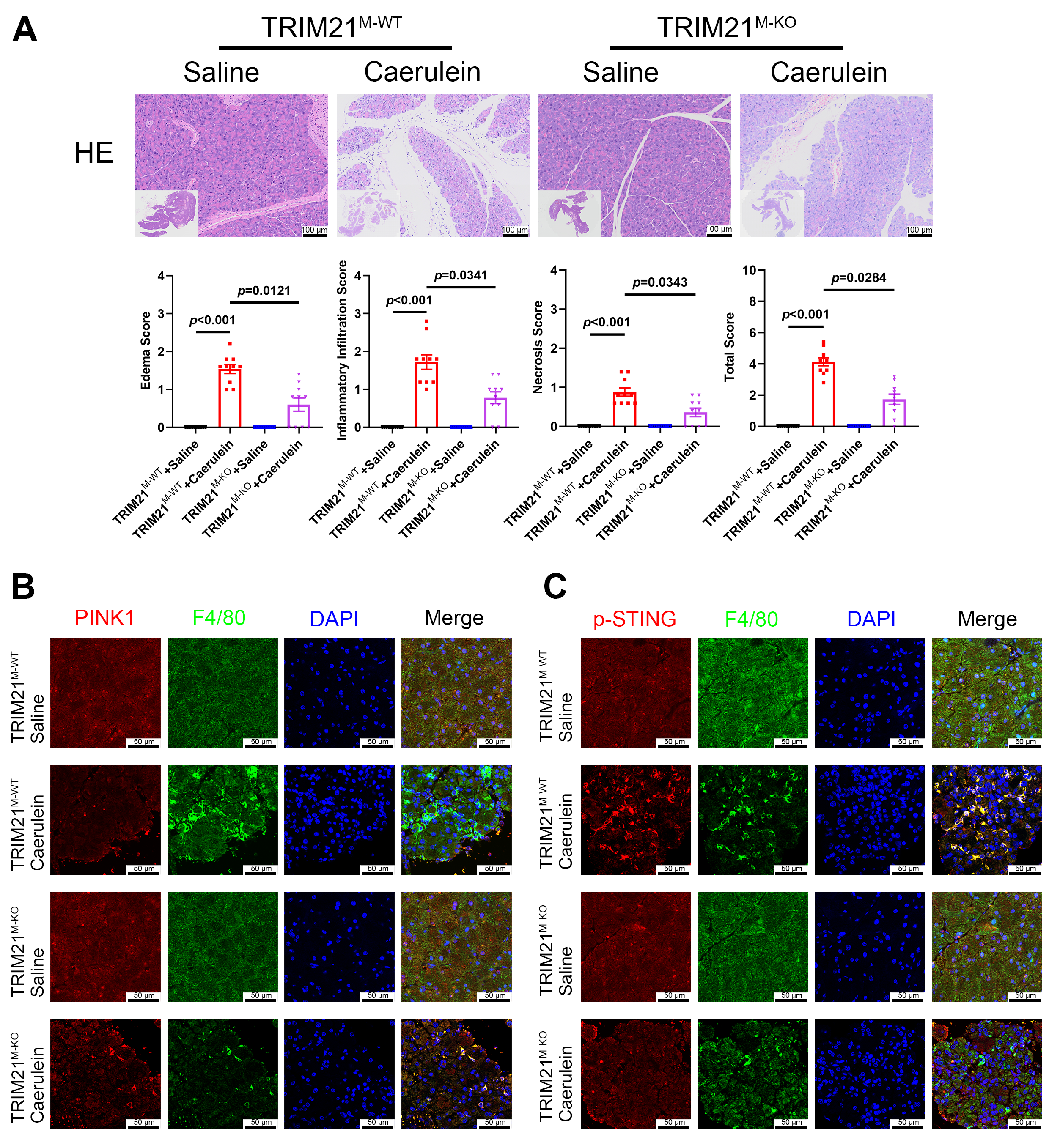


**Figures S5.** Macrophage *Trim21* deficiency alleviated pancreatic injury by suppressing mitophagy-driven cGAS-STING activation during caerulein-induced AP. (A-C) *Trim21^M-KO^* and *Trim21^M-WT^* mice were treated with either saline or caerulein for 12 hours (*Trim21^M-WT^* + Saline, *Trim21^M-WT^* + Caerulein, *Trim21^M-KO^* + Saline, *Trim21^M-KO^* + Caerulein). (A) H&E staining (upper panel) and histopathological scoring (lower panel) of pancreatic tissue (n = 10 per group). (B, C) Representative co-immunofluorescence images of PINK1 (red; B) or p-STING (red; C) with the macrophage marker F4/80 (green) in pancreatic tissues. Cell nuclei were stained with DAPI (scale bar: 50 μm; n = 5 per group). Data are expressed as mean ± SEM (A), and statistical analyses were conducted using Kruskal-Wallis test with Dunn's post hoc test (A).

**
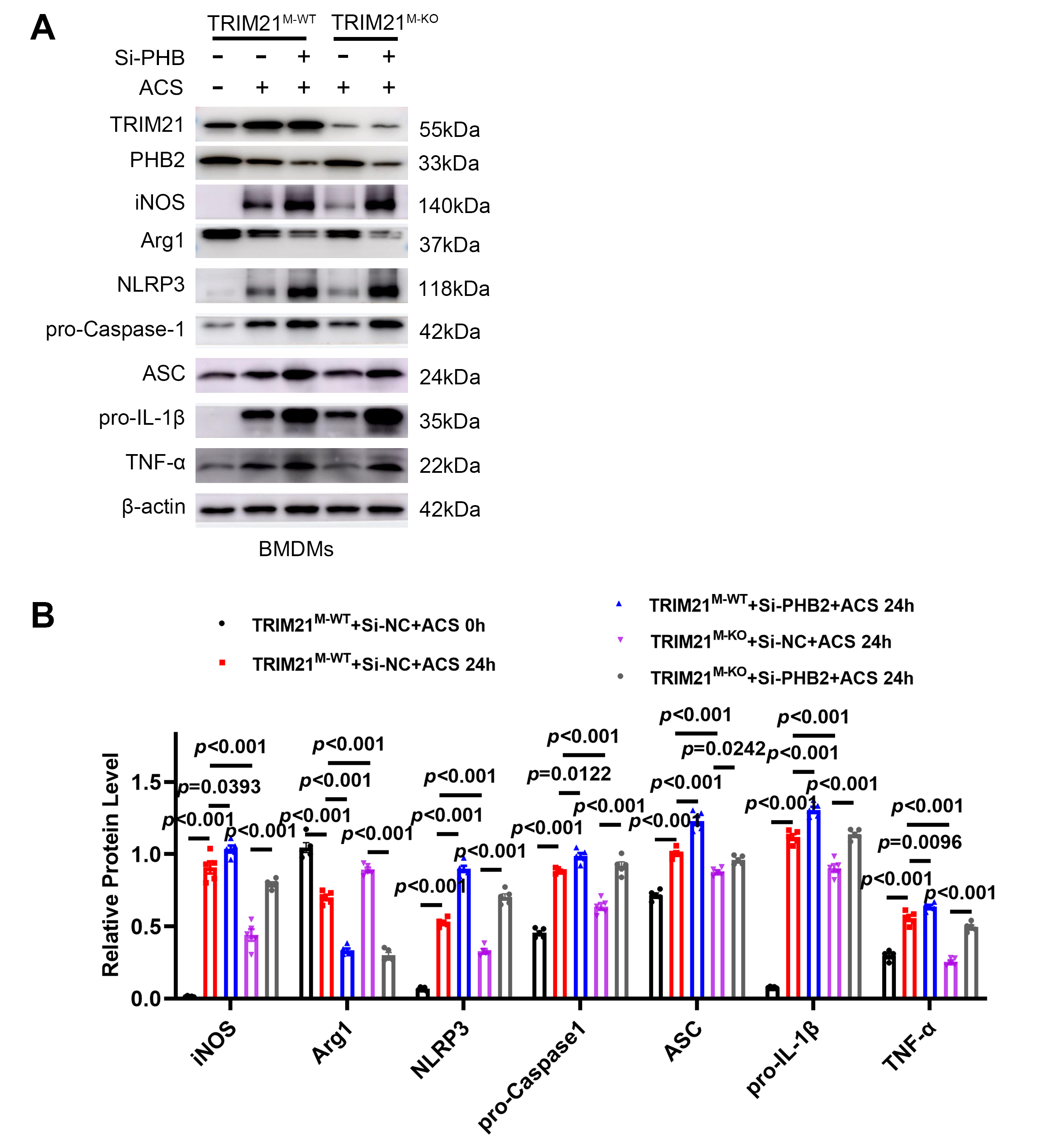
**

**Figures S6.** The effect of combined macrophage *Trim21* deficiency and *Phb2* knockdown on macrophage phenotype and inflammatory proteins in ACS-treated BMDMs. (A and B) Representative Western blot images and quantiﬁcation of phenotype-specific proteins (iNOS and Arg1) and inflammatory proteins (NLRP3, pro-caspase-1, ASC, pro-IL-1β, TNF-α) in BMDMs from *Trim21^M-WT^* or *Trim21^M-KO^* mice following transfection with si-*Phb2* and subsequent 12-hour ACS treatment (n = 5). Data expressed as mean ± SEM (B). Statistical analyses were performed using ANOVA followed by Tukey HSD post hoc test (B).


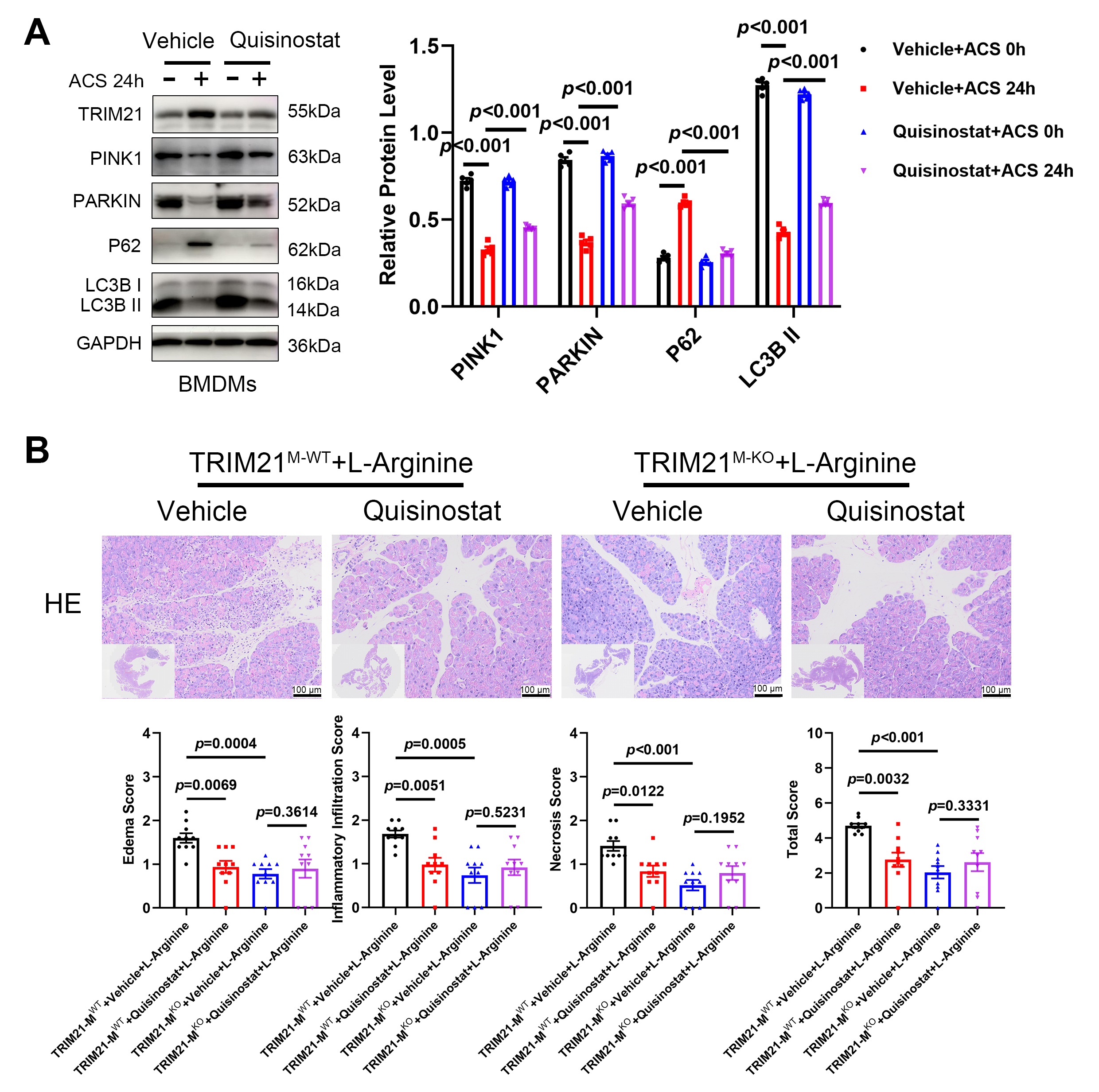


**Figures S7.** Quisinostat ameliorated AP in a TRIM21-dependent manner. (A) Representative Western blot images (left panel) and quantiﬁcation (right panel) of mitophagy-associated protein levels (PINK1, Parkin, p62, and LC3B) in BMDMs from *Trim21^M-WT^* mice pretreated with quisinostat and then stimulated with ACS for 12 h (n = 5). (B) *Trim21^M-KO^* and *Trim21^M-WT^* mice pretreated with vehicle or quisinostat intraperitoneally received L-arginine for 72 hours (*Trim21^M-WT^* + Vehicle + L-arginine, *Trim21^M-WT^* + Quisinostat + L-arginine, *Trim21^M-KO^* + Vehicle + L-arginine, *Trim21^M-KO^* + Quisinostat + L-arginine; n = 10 per group). (B) H&E staining (upper panel) and histopathological scoring (lower panel) of pancreatic tissue. Data are expressed as mean ± SEM (A and B), and statistical analyses were conducted using ANOVA followed by Tukey HSD post hoc test (A) and Kruskal-Wallis test with Dunn's post hoc test (B).

# Supporting Information Tables

**Table S1.** Baseline profiles of ELISA-tested cohorts: AP patients vs. age-matched health controls

| Variables | Health Control  (n=29) | MAP  (n=41) | MSAP  (n=37) | SAP  (n=23) | *H*/*F*/χ² | *P*-value |
| --- | --- | --- | --- | --- | --- | --- |
| Age [years] | 39.0 (36.0, 50.0) | 51.0 (41.0, 57.0) | 44.0 (36.0, 56.0) | 48.0 (37.5, 56.0) | 7.39 | 0.0605^a)^ |
| Male (n, %) | 13 (45) | 18 (44) | 20 (54) | 8 (35) | 2.20 | 0.5310^c)^ |
| BMI [kg/m^2^] | 21.48 (20.02, 24.17) | 22.90 (21.69, 24.84) | 23.64 (21.34, 25.84) | 25.69 (22.46, 27.60) | 11.94 | 0.0076^a)^ |
| Smoking (n, %) | 6 (21) | 11 (27) | 9 (24) | 4 (17) | 0.87 | 0.8328^c)^ |
| Drinking (n, %) | 3 (10) | 9 (22) | 6 (16) | 6 (26) | 2.61 | 0.4552^c)^ |
| Amylase [U/L] | 39.83 ± 5.73 | 382.80 ± 112.55 | 381.22 ± 115.89 | 472.17 ± 98.38 | 368.86 | <0.001^b)^ |
| TC [mmol/L] | 5.63 (5.45, 5.77) | 7.49 (6.69, 8.30) | 6.99 (6.40, 7.80) | 9.71 (7.81, 11.82) | 51.27 | <0.001^a)^ |
| FBG [mmol/L] | 4.90 (4.40, 5.50) | 6.90 (5.20, 8.70) | 6.60 (5.50, 8.60) | 10.30 (8.60, 12.95) | 45.39 | <0.001^a)^ |
| Hypertension (n, %) | 4 (14) | 10 (24) | 6 (16) | 4 (14) | 1.52 | 0.6780^c)^ |
| Diabetes Mellitus (n, %) | 5 (17) | 8 (20) | 5 (14) | 7 (30) | 2.71 | 0.4380^c)^ |

AP, Acute pancreatitis; MAP, Mild acute pancreatitis; MSAP, Moderately severe acute pancreatitis; SAP, Severe acute pancreatitis; BMI, Body mass index; TC, Total cholesterol; FBG, Fasting blood glucose. ^a)^Kruskal-Wallis test; ^b)^Welch's ANOVA; ^c)^Chi-squared test.

**Table S2 and Table S3 are provided as Microsoft Excel files and have been uploaded separately as Supporting Information.**

**Table S4.** Experimental materials

| Reagent or resource | Source | Identifier |
| --- | --- | --- |
| Antibodies for Western Blot | | |
| TRIM21 | Abcam | ab207728 |
| PHB2 | Proteintech | 12295-1-AP |
| iNOS | Abcam | ab178945 |
| Arg1 | Abcam | ab233548 |
| IL-1β | Proteintech | 66737-1-Ig |
| TNF-α | Proteintech | 60291-1-Ig |
| NLRP3 | Abcam | ab263899 |
| pro-caspase-1 | Abcam | ab179515 |
| ASC | Abcam | ab309497 |
| cGAS | Cell Signaling Technology | 31659T |
| p-TBK1 | Cell Signaling Technology | 5483T |
| TBK1 | Cell Signaling Technology | 3504T |
| p-NF-κB | Proteintech | 82335-1-RR |
| NF-κB | Proteintech | 80979-1-RR |
| p-IRF3 | Cell Signaling Technology | 29047T |
| IRF3 | Cell Signaling Technology | 4302T |
| p-STING | Cell Signaling Technology | 72971T |
| STING | Cell Signaling Technology | 50494T |
| PINK1 | Proteintech | 23274-1-AP |
| Parkin | Proteintech | 14060-1-AP |
| p62 | Abcam | ab109012 |
| LC3B | Abcam | ab229327 |
| His | Abcam | ab1220 |
| Flag | Abcam | ab205606 |
| MYC | Abcam | ab9106 |
| Ubiquitin | Abcam | ab134953 |
| β-actin | Abcam | ab8226 |
| GAPDH | Abcam | ab8245 |
| Goat anti-rabbit IgG-HRP | Cell Signaling Technology | 7074S |
| Goat anti-mouse IgG-HRP | Cell Signaling Technology | 7076S |
| Mouse Anti-Rabbit IgG LCS | AmyJet | AMJ-AB2017 |
| Antibodies for Co-immunoprecipitation (Co-IP) Assay | |  |
| TRIM21 | ABclonal | A1957 |
| PHB2 | Proteintech | 12295-1-AP |
| MYC | Abcam | ab9106 |
| Flag | Abcam | ab205606 |
| His | Abcam | ab1220 |
| Antibodies for Immunochemical Staining | | |
| TRIM21 | Abcam | ab207728 |
| IL-1β | Proteintech | 66737-1-Ig |
| TNF-α | Proteintech | 60291-1-Ig |
| Reagents for Immunofluorescent Staining | | |
| TRIM21 | Proteintech | 67136-1-Ig |
| PHB2 | Proteintech | 12295-1-AP |
| PINK1 | Proteintech | 23274-1-AP |
| p-STING | Cell Signaling Technology | 62912T |
| F4/80 | Biolegend | 123120 |
| MPO | Abcam | ab208670 |
| TOMM20 | Abcam | ab78547 |
| TFAM | ABclonal | A3173 |
| dsDNA | Abcam | ab27156 |
| Ad-mCherry-GFP-LC3B | Beyotime | C3011 |
| Primer Sequences | | |
| m-*Trim21* | F: 5′-CTATGAGGCTGGCGTTGTCT-3′ | |
|  | R: 5′-GCTTTAGAGGCGCTGCATTT-3′ | |
| m-*Trim8* | F: 5′-CCCCACCCTGCTTAACCCTT-3′ | |
|  | R: 5′-TTGGCACCCAGTCCTCTCAA-3′ | |
| m-*Trim37* | F: 5′-GGTACAGCCCACACTACCTG-3′ | |
|  | R: 5′-ATGTGAATCTTCATCGGGGG-3′ | |
| m-*Trim41* | F: 5′-CTGACACTGGACCCTGACAC-3′ | |
|  | R: 5′-GATGGGTTGATTCACGGGCA-3′ | |
| m-*Trim65* | F: 5′-CAATGGCAAATCTCAGCGCC-3′ | |
|  | R: 5′-ACAGAGTAAGGGTCCTCCCC-3′ | |
| m-*Phb2* | F: 5′-ATCCGTGTTCACCGTGGAAG-3′ | |
|  | R: 5′-CCCGAATGTCATAGATGATGGG-3′ | |
| m-*MtDNA Dloop* | F: 5′-AATCTACCATCCTCCGTGAAACC-3′ | |
|  | R: 5′-TCAGTTTAGCTACCCCCAAGTTTAA-3′ | |
| m-*18s* | F: 5′-GTAACCCGTTGAACCCCATT-3′ | |
|  | R: 5′-CCATCCAATCGGTAGTAGCG-3′ | |
| m-*Gapdh* | F: 5′-TGTCTCCTGCGACTTCAACA-3′ | |
|  | R: 5′-GGTGGTCCAGGGTTTCTTACT-3′ | |
